# The proteome, not the transcriptome, predicts that oocyte superovulation affects embryonic phenotypes in mice

**DOI:** 10.1038/s41598-021-03054-9

**Published:** 2021-12-09

**Authors:** Leila Taher, Steffen Israel, Hannes C. A. Drexler, Wojciech Makalowski, Yutaka Suzuki, Georg Fuellen, Michele Boiani

**Affiliations:** 1 Institute of Biomedical Informatics, Graz University of Technology, Stremayrgasse 16/I, 8010 Graz, Austria; 2 Max Planck Institute for Molecular Biomedicine, Roentgenstrasse 20, 48149 Muenster, Germany; 3 Institute for Biostatistics and Informatics in Medicine and Aging Research (IBIMA), Rostock University Medical Center, Ernst-Heydemann-Strasse 8, 18057 Rostock, Germany; 4 Institute of Bioinformatics, Faculty of Medicine, University of Münster, Niels Stensen Str. 14, 48149 Münster, Germany; 5 Department of Medical Genome Sciences, Graduate School of Frontier Sciences, University of Tokyo, Kashiwa, Chiba 277-8562 Japan

**Keywords:** Developmental biology, Embryology, Proteomics

## Abstract

Superovulation is the epitome for generating oocytes for molecular embryology in mice, and it is used to model medically assisted reproduction in humans. However, whether a superovulated oocyte is normal, is an open question. This study establishes for the first time that superovulation is associated with proteome changes that affect phenotypic traits in mice, whereas the transcriptome is far less predictive. The proteins that were differentially expressed in superovulated mouse oocytes and embryos compared to their naturally ovulated counterparts were enriched in ontology terms describing abnormal mammalian phenotypes: a thinner zona pellucida, a smaller oocyte diameter, increased frequency of cleavage arrest, and defective blastocyst formation, which could all be verified functionally. Moreover, our findings indicate that embryos with such abnormalities are negatively selected during preimplantation, and ascribe these abnormalities to incomplete ovarian maturation during the time of the conventional superovulation, since they could be corrected upon postponement of the ovulatory stimulus by 24 h. Our data place constraints on the common view that superovulated oocytes are suitable for drawing general conclusions about developmental processes, and underscore the importance of including the proteins in a modern molecular definition of oocyte quality.

## Introduction

The term ‘superovulation’ was introduced in 1927^1,2^. It has become the epitome for generating oocytes for experimental and molecular embryology in mice, in greater numbers than natural ovulation, by stimulation with exogenous gonadotropins. Studies on both superovulation and pregnancy in mice set the stage for assisted human reproduction^3–5^. The superovulation of mice still relies on the administration of equine and human chorionic gonadotropin, eCG and hCG, about 48 h apart^6^. There has been many studies to illuminate developmental processes using superovulated oocytes, in lieu of naturally ovulated oocytes. One could say provocatively that molecular mouse embryology is an embryology of superovulation. The reason for preferring the superovulated oocytes is one of convenience: the time of oocyte collection can be scheduled, and fewer donor animals are needed, thereby contributing to implementing the principles of reduction, replacement and refinement (3 Rs) in animal research^7^. The studies suggested that the superovulated oocytes were either equivalent or not equivalent to naturally ovulated oocytes. These conflicting conclusions were based on heterogeneous epigenetic and rare transcriptomic data. We hypothesized that proteomic data may provide a more robust answer since gene transcription is silenced in late oogenesis, and the initial stages of embryogenesis are controlled by maternal proteins^8–10^.

Oocyte quality aberrations associated with the gonadotropins eCG and hCG were not observed equally across mouse studies. Superovulated oocytes had reduced ability for endocytosis^11^ and derivative blastocysts had fewer microvilli^12^ than naturally ovulated counterparts, but ultrastructural differences were otherwise unimpressive. Some studies reported an increase of the rate of embryo aneuploidy and fetal malformation by use of gonadotropins^13–17^, while others contradicted these findings^4,18,19^. Some studies linked the superovulation to a direct effect on the acquisition of DNA methylation in oocytes^20–24^, including epimutations^25^, while other studies found no such effect^26,27^. In two studies that correlated oocyte diameters to oocyte maturity and DNA methylation^28^, one study suggested that superovulation results in smaller immature oocytes that have not completed the acquisition of DNA methylation^29^, whereas the other study found that oocyte diameters do not differ between superovulation and natural ovulation^27^. There were confounders in most of the studies above: the age and strain of mice varied, so did the time spent by the oocytes in the genital tract of the stimulated female (interval between superovulation and collection for study). There has been a concern that superovulation may create an unfavorable somatic (endocrine) environment for the developing embryo in either the oviduct^30,31^ or the uterus^32–38^; the latter would lead to reduced endometrial receptivity and, thereby, reduced blastocyst implantation, with later consequences for placental function^39^. Unlike the interval between superovulation and oviductal retrieval, the interval between eCG and hCG has been neglected in mice, with only two studies that reached different conclusions^40,41^. However, it is evident that the accumulation of gene products in oocytes is a function of the time spent in the follicles until the ovulatory stimulus (including the time between eCG and hCG in the case of superovulation).

The overarching aim of our study was to clarify the mechanism of the superovulation effect on oocyte quality in adult mice, drawing from a type of data not previously used in a context of superovulation *vs.* natural ovulation. Large-scale protein studies comparing superovulated and naturally ovulated mouse oocytes are lacking, despite hints that protein synthesis in oocytes^42,43^ and maternal proteins required for imprint maintenance after fertilization^44^ may be disturbed by gonadotropins. Transcriptome analysis has only been used once to compare superovulated and naturally ovulated mouse oocytes^45^, with inconspicuous findings. No tandem studies of the transcriptome and proteome have been devoted to the comparison between the gene expression profiles of superovulated and naturally ovulated mouse oocytes and derivative embryos so far, in a way that the two groups differ solely by the superovulation. Building on our record of proteome analysis of oocytes and embryos^46–51^, we conducted a comparative proteome vs. transcriptome analysis of the two groups. The predictions of the gene expression analysis were cross-checked with the occurrence of relevant phenotypes during pre- and post-implantation development. The protein – but not the transcript – data established that superovulated mouse oocytes are not equivalent to their natural counterparts: the extent of preovulatory development prior to the ovulatory stimulus is decisive. These results underscore the importance of including the proteins in a modern molecular definition of oocyte quality, and call for caution when relying on superovulated oocytes to draw general conclusions about developmental processes.

## Results

### Differences between superovulated oocytes and embryos, and their naturally ovulated counterparts, are more pronounced in the proteome than in the transcriptome

**Figure 1:**
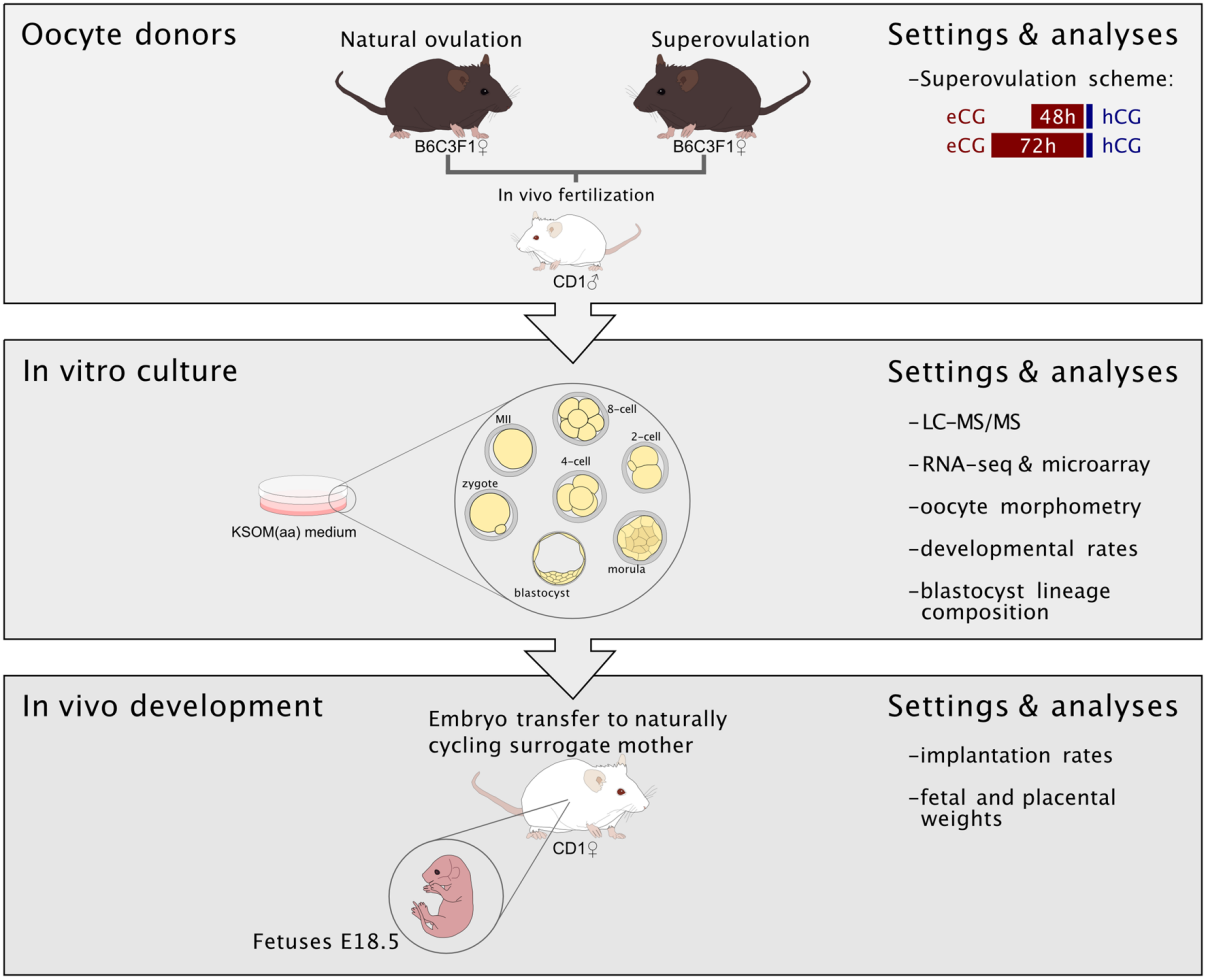
Experimental design. B6C3F1 females aged 8–10 weeks were injected with gonadotropins (superovulation) or with vehicle (natural ovulation) and caged with vasectomized or stud CD1 males to obtain MII or fertilized oocytes, respectively. These were removed at 8 a.m. on the day of the vaginal plug, pooled, and equalized by in vitro culture (IVC). Embryos were sampled in vitro at defined stages for molecular analysis or transplanted to naturally cycling females to generate fetuses. eCG, equine chorionic gonadotropin; hCG, human chorionic gonadotropin; IVC, in vitro culture; LC–MS/MS, liquid chromatography with tandem mass spectrometry; MII, metaphase II.

We quantified and compared the proteome and the transcriptome of hybrid inbred mouse oocytes and preimplantation embryos in two groups: i) ‘superovulation’ (treatment) and ii) ‘natural ovulation’ (control). The dose of gonadotropins used in our mice was chosen to preserve the units-to-body weight ratio of the commonly used 5 I.U. eCG and hCG given at the age of 4 weeks, when mice weigh 10–15 g^6^: therefore, our mice were given 10 I.U. at the age of 8–10 weeks, when they weigh 20–25 g. Superovulation resulted in 26.9 ± 10.3 oocytes per female, compared to 7.9 ± 1.6 oocytes of the natural ovulation (total oocytes = 538 and 173, respectively). We removed the oocytes from the oviducts in each of the groups either before or after fertilization in vivo, instead of leaving them in the genital tract^39,52,53^, thereby removing the developmental confounder of the maternal environment. Oocytes of each group were pooled and equalized by culturing in potassium (K) simplex optimized medium containing amino acids (KSOM(aa)) (Fig. 1), thus, enabling a follow-up of the fertilized oocytes during preimplantation (2-cell, 4-cell, 8-cell, morula, blastocyst; Supplementary Fig. S1). We collected two biological replicates for proteomics and transcriptomics analyses for all developmental stages after the appropriate time spent in culture. The in vitro culture was a valid choice, since blastocysts flushed from the uterus after superovulation exhibited poor morphology, as exemplified by the degree of cavity expansion (Supplementary Fig. S2). This difference attests to the influence of undefined cues emanating from the genital tract of stimulated females and the importance – in discovery studies – of eliminating the confounder of the maternal environment.

Protein expression was measured by liquid chromatography with tandem mass spectrometry (LC–MS/MS) analysis of zona-free specimens, as per our established analysis pipeline^46–51^. The zona-free cells were concentrated in a 2–3 μL volume of protein-free medium (Materials and methods), as shown exemplarily for the oocytes (Supplementary Fig. S1, ‘pellet’), before adding 10 μL of lysis buffer. Protein abundance was quantified using the intensity-based absolute quantification (iBAQ) algorithm (Materials and methods), which provides adimensional values that are proportional to the *molar fraction of* the proteins in the cell. We detected a total of 6444 proteins across all samples (Supplementary Table S1). We sampled aliquots from the specimens collected for proteomics. The aliquots were subjected to RNA-seq and microarray analysis, returning a transcriptome of 20,887 transcripts (Supplementary Table S2).

**Figure 2:**
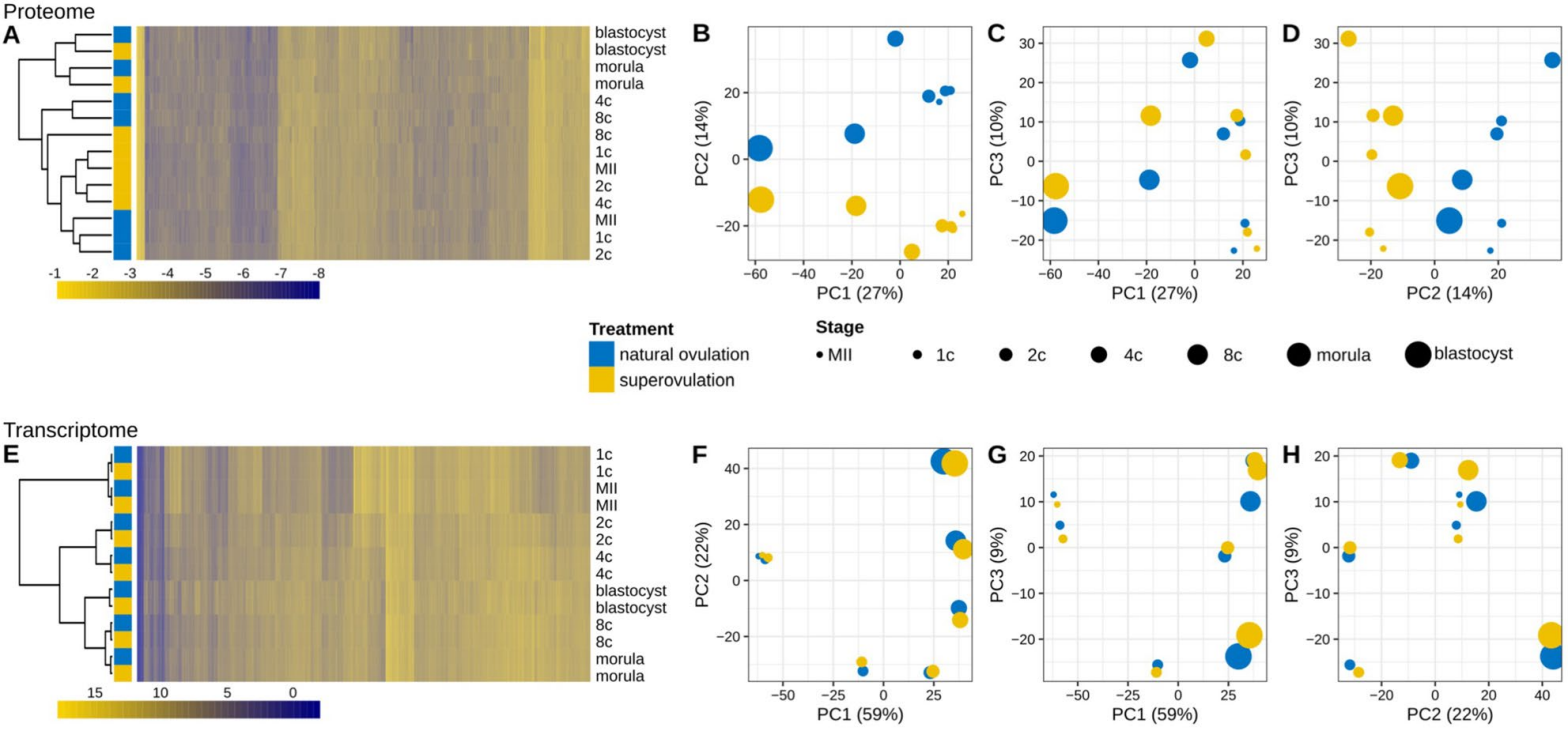
Expression analysis of the constitutively expressed (n = 2844) proteomes and their transcriptomic counterparts. (**A**, **E**) Hierarchical clustering of the proteome (**A**) and the transcriptome (**E**). Samples were clustered with the complete linkage algorithm, using a distance matrix based on the Pearson correlation coefficient computed between the expression values of the proteins. Proteins/transcripts are sorted according to pairwise Euclidean distances. (**B**–**D** and **F**–**H**) Principal component analysis (PCA). Each data point corresponds to a stage and treatment group. The first three principal components (PCs) of the data are represented. The PCA of the proteome resolves the superovulation and the natural ovulation groups in the 2nd component, whereas the PCA of the transcriptome resolves the two groups in the 6th component (not shown). Samples were derived from pools of 200–250 (proteome) and 210 (transcriptome) oocytes or embryos in duplicate for each treatment.

Among the 6444 proteins, 2844 were detected in both treatment groups at all stages and in at least one replicate and were, therefore, considered “constitutively expressed” (Supplementary Table S3). Hierarchical clustering of the expression profile of these 2844 proteins separated the samples distinctly into two clusters (Fig. 2A): a cluster comprising the oocyte and 1-, 2-, 4-, and 8-cell stages, and one comprising the morula and blastocyst stages. While the samples in the latter were clustered by stage, with superovulation and natural ovulation samples clustering together, the samples in the former were clustered according to a different pattern. Those samples were separated into four clusters consisting of exclusively superovulation or natural ovulation samples. This was in stark contrast to the hierarchical clustering of the corresponding transcripts, which showed all samples clustered by stage, with superovulation and natural ovulation samples clustering together (Fig. 2E). These findings were robust, irrespective of whether we considered the set of constitutively expressed genes (Fig. 2) or the parent sets of 6444 proteins and 20,887 transcripts (Supplementary Fig. S3). The hierarchical clustering results were consistent with principal component analysis (PCA). Regarding the proteome, the first principal component (PC1, ~ 27% of the variance) predominantly captured the developmental trajectory of the embryos (Fig. 2B), confirming our previous study^47^. The second principal component (PC2, ~ 14% of the variance) distinguished between superovulation and natural ovulation oocytes and derivative embryos (Fig. 2C). By contrast, the PCA of the transcriptome (Fig. 2F-H) revealed a much smaller effect of the treatment, which was only primarily captured by the sixth principal component (PC6, ~ 1% of the variance).

These results reveal differences between the gene expression profiles of superovulated oocytes and derivative embryos and their naturally ovulated counterparts when compared in the same in vitro environment. This molecular distinction is more pronounced in the proteome than in the transcriptome, delineating a gonadotropin-sensitive proteome. Since proteomics has not been used before to compare superovulation to natural ovulation, it follows that superovulation may affect developmental processes in unimagined ways.

### A distinctive gene ontology signature in the altered proteome of superovulated oocytes

**Figure 3:**
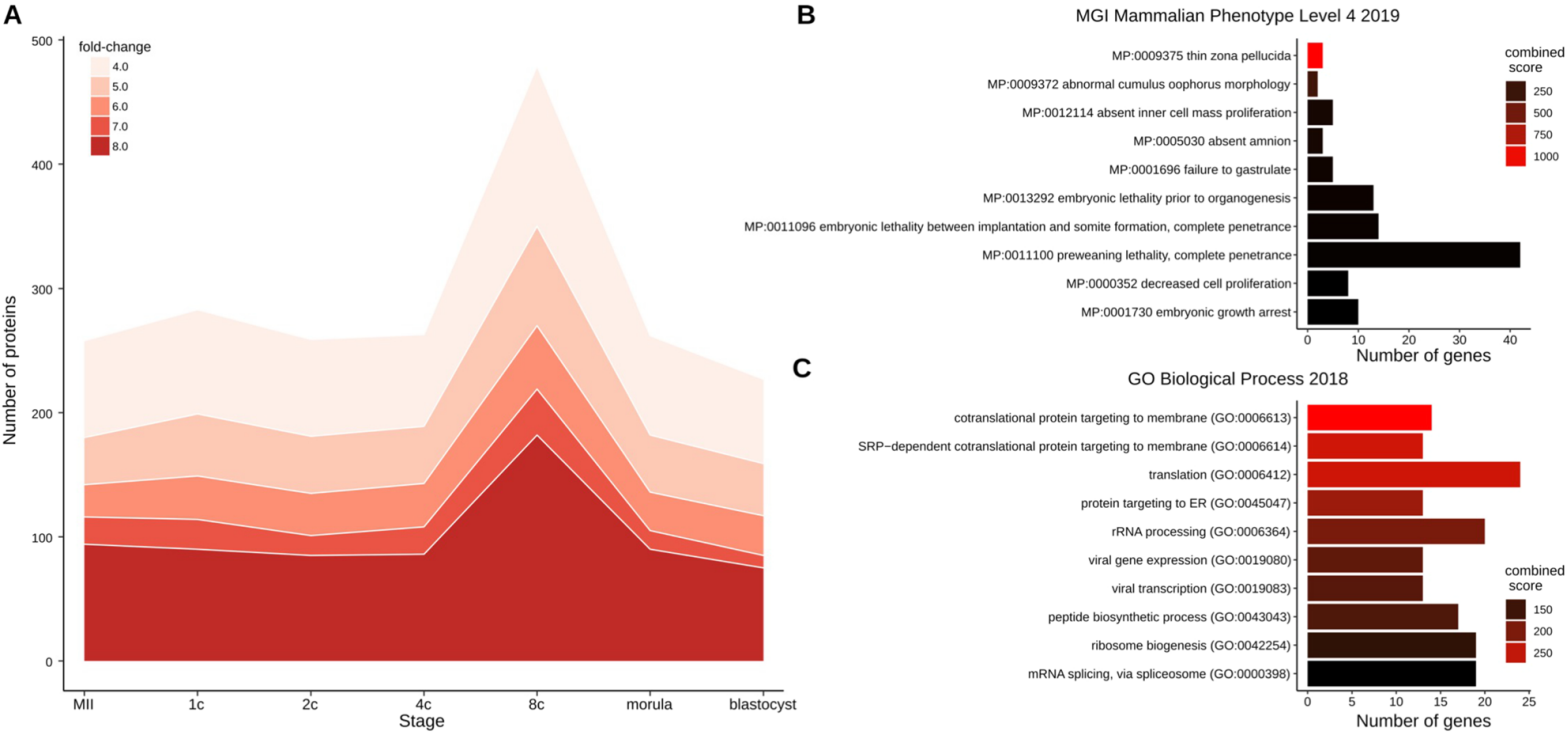
Numbers of gonadotropin-sensitive proteins and their functional enrichment analysis. (**A**) Stage-specific number of proteins exhibiting a fold-change greater than or equal to 4, 5, 6, 7, or 8 (see scale) between the superovulation and natural ovulation groups. (**B**, **C**) Functional enrichment analysis of proteins expressed differentially using Enrichr. Only the ten terms with the smallest P values are shown. Terms are sorted according to their combined scores, which are computed by taking the logarithm of the P-values and multiplying them by the z-scores of the deviation from the ranks expected. (**B**) Gene ontology terms of the altered proteome in the ontology ‘Mammalian Phenotype’. (**C**) Gene ontology terms of the altered proteome in the ontology ‘Biological process’.

We examined the fold-changes of the expression values of the 2844 proteins between the superovulation and natural ovulation group to gain a general impression of how the gonadotropin-sensitive proteome fluctuates as a function of the developmental stage. Accordingly, we counted the number of proteins exceeding a particular fold-change (Fig. 3A). Irrespective of the fold-change, the number of altered proteins were greatest at the 8-cell stage and smallest in the blastocysts – consistent with a prior removal of the unfit or unviable embryos by natural selection. The overall profile suggests that specific proteins may be systematically up- or downregulated across the entire developmental series in response to superovulation. We, therefore, examined this possibility.

A total of 278 of 2844 (~ 10%) proteins were differentially expressed for all stages upon superovulation (Wilcoxon test; Supplementary Table S4; Materials and methods). The abundance of these 278 proteins is systematically higher compared to that of the parent proteome, as determined from the probability density functions of riBAQ values (Supplementary Fig. S4). Of the 278 proteins, 115 were also differentially expressed in excess of twofold (56 up, 59 down, Supplementary Table S4).

Functional enrichment analysis of the 278 proteins expressed differentially revealed several terms in the Mammalian Phenotype (MP) Ontology that are relevant for pre- and post-implantation embryos and prompted additional experiments (see next section), including “MP:0009375 thin zona pellucida” (ZP), “MP:0001730 embryonic growth arrest,” “MP:0012114 absent inner cell mass proliferation,” and “MP:0011100 preweaning lethality, complete penetrance” (Fig. 3B; Supplementary Table S4). The proteins expressed differentially also featured several terms in the gene ontology ‘Biological process’ (Fig. 3C), with a stronger link to translation than to transcription. While this result may be unsurprising given the fact that we are analyzing proteomic data, it is also clear that protein activities enable transcription by, for example, RNA polymerases. The fact that RNA- and transcription-related functions are weakly represented among the proteins expressed differentially is entirely consistent with the results of the transcriptome analysis. Indeed, no transcripts were found expressed differentially (Supplementary Table S5), in contrast to the proteome. This was also the case for an additional transcriptomic dataset which was based on a different platform (Affymetrix; GEO number GSE110599) but was derived from the same specimens used for the RNA-seq analysis. Therefore, compared to their naturally ovulated counterparts, superovulated oocytes and embryos can be resolved at the protein level at a greater degree than at the transcript level. This is consistent with our previous findings^47^.

We next undertook a series of phenotypic analyses to test the robustness of the gene ontology signature of the perturbed proteome.

### Phenotypic traits substantiate the functional relevance of the altered proteome

**Figure 4:**
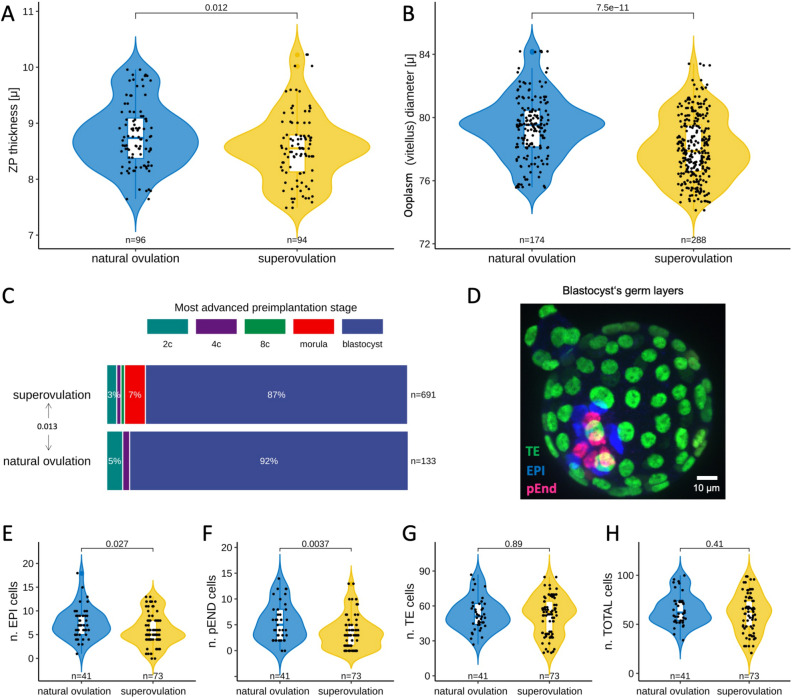
Preimplantation phenotypes of superovulated and naturally ovulated mouse oocytes. (**A**) The thickness of the ZP. Each data point corresponds to one oocyte. Wilcoxon test. (**B**) The diameter of the oocyte proper i.e. excluding ZP and perivitelline space (vitellus). Each data point corresponds to one oocyte. Wilcoxon test. (**C**) The most advanced preimplantation stage reached in KSOM(aa) by the embryos of superovulated and naturally ovulated oocytes. Note that the scoring started from the 2-cell stage, since arrested 1-cells could be either oocytes which were not fertilized, or fertilized oocytes which failed to cleave. χ^2^ test. (**D**) Representative image of the triple immunostaining used to assign the blastocyst cells to the primary germ layers. (**E**–**H**) the cell numbers counted in the primary germ layers of blastocysts formed in KSOM(aa) by superovulated and naturally ovulated oocytes. Each data point corresponds to one blastocyst. Wilcoxon test. n, number of; TE, trophectoderm; EPI, epiblast; pEND, primitive endoderm; ZP, zona pellucida.

The functional enrichment analysis of the 278 proteins expressed differentially pointed at the ZP. This is represented in our dataset by the three proteins Zp1, Zp2, and Zp3, which account for the complete protein composition of the murine ZP. These proteins were systematically less abundant in superovulated oocytes and derivative embryos compared to their naturally ovulated counterparts. By contrast, no change was observed in the abundances of housekeeping genes (Supplementary Table S6). Differences in the abundances of ZP proteins appear to be an unexpected finding, given that the specimens of the proteomic analysis had been manually deprived of the ZP (Materials and methods). However, it is pertinent to recall that ZP proteins are synthesized in the cytoplasm; therefore, the alterations observed are presumably associated with the cytoplasmic precursors of the ZP proteins. In order to confirm this, we generated additional samples of ZP-free superovulated oocytes (Supplementary Fig. S1) and compared them to ZP-enclosed oocytes via Western blot analysis, using a validated Zp3 antibody^54^ (Materials and methods). This assay recognized a strong single band of the correct molecular weight regardless of whether the ZP had been removed or not, thereby showing that the bulk of Zp3 is intracellular (Supplementary Fig. S1, S7). The ZP proteins are translocated into the endoplasmic reticulum, secreted, and assembled into fibrils outside the cell exclusively in growing oocytes. Therefore, our observations suggest that the oocytes had progressed unequally far into oogenesis when they were recruited for ovulation. In order to test this possibility, we left the ZP in place and measured its thickness and the diameter of the ooplasm (excluding the zona and perivitelline space) of freshly isolated, cumulus cell-free oocytes taken in parallel for the superovulation and natural ovulation groups (Materials and methods). For brevity, the ooplasm without the ZP is also referred to as an ‘oocyte proper’ or ‘vitellus.’ Indeed, superovulated oocytes exhibited a thinner ZP compared to their naturally ovulated counterparts (median 8.5 vs. 8.7 μm; interquartile range 8.1–8.8 μm vs. 8.3–9.1 μm; p = 0.012, Wilcoxon test; Fig. 4A). Moreover, we inspected the diameter of the ooplasm since the oocyte diameter increases with the ZP thickness^55^. We derived the diameter from the perimeters drawn manually along the oolemma in the equatorial section of the oocytes (Materials and methods) since oocytes are approximately, but not exactly, spherical in shape. We found that the diameter of the oocyte proper was smaller for the superovulated oocytes compared to that of their naturally ovulated counterparts (78.1 ± 2.0 μm vs. 79.4 ± 2.0 μm, p < 0.0001, Wilcoxon test; Fig. 4B). This dimensional difference in diameter accounts for a volume difference of 4.7%, as calculated by applying spherical shape approximation (superovulated oocytes: 250 picoliters; naturally ovulated oocytes: 262 picoliters).

Another hit of the functional enrichment analysis, the MP term “embryonic growth arrest,” was represented in our dataset by ten proteins expressed differentially (Emg1, Kdm1a, Hdac1, Cox17, Sumo2, Sbds, Cul1, Lamtor1, Pelo, and Cops5; Supplementary Table S6). In order to assess this term functionally, we scored the proportions of embryos arrested at each preimplantation stage, starting from the 2-cell stage, to ensure that the oocyte had been fertilized (unfertilized oocytes would not cleave). The proportion of embryos arrested at or past the 2-cell stage is expected to be negligible if the IVC system is adequate (e.g. KSOM(aa)). The vast majority of naturally ovulated oocytes consistently progressed to blastocyst. By contrast, embryos of superovulated oocytes were more often arrested directly prior to the blastocyst stage (p = 0.013, χ^2^ test; Fig. 4C), with the greatest rate of loss at the morula stage. This is reminiscent of the observations made when embryos were flushed from the uterus after mating, following to natural ovulation *vs.* superovulation: the former had the most losses at the beginning of development, while the latter had them toward the end of preimplantation and featured blastocysts of poor morphology (Supplementary Fig. S2). Thus, unfit embryos of superovulated oocytes seemed to reach further, i.e. to be selected later (morula-arrest), compared to natural ovulation (progression to blastocyst if not 2-cell arrest).

A third hit of the functional enrichment analysis, MP term “absent inner cell mass proliferation,” was represented in our dataset by five proteins expressed differentially (Sbds, Pelo, Cdc73, Cops5, and Dab2; Supplementary Table S6). Since this term should reflect a direct impact on the number of cells found in the inner cell mass, and proteins Dab2 and Cops5 are lineage markers^56^, we counted those cells making a further distinction between the subcompartments of the inner cell mass (i.e. epiblast and primitive endoderm cells). We counted the remaining blastocyst lineage, namely the trophectoderm, as a control. We relied on our immunostaining protocol to identify the cells in the three compartments (Fig. 4D; Materials and methods). The two subpopulations of the inner cell mass had fewer cells in the superovulation group (primitive endoderm, p = 0.027; epiblast, p = 0.004, Wilcoxon test; Fig. 4E,F), while the trophectodermal and total cell counts were indistinguishable between blastocysts of the superovulation and natural ovulation groups (Fig. 4G,H). Furthermore, the depletion was not merely a reduction, but also featured cases of either no cells or just one cell in the endoderm compartment (p = 0.002; Fisher’s exact test). These findings are informative because the primitive endoderm and epiblast can interconvert via FGF signaling^57^, and a sufficient number of cells is needed in the epiblast in order for the blastocysts to be competent for fetal development^58,59^. It is, therefore, conceivable that cells might need to be present in sufficient numbers in not only the epiblast but also the primitive endoderm. Hence, we set out to examine how the embryos of the superovulation and natural ovulation group fare at post-implantation.

**Figure 5:**
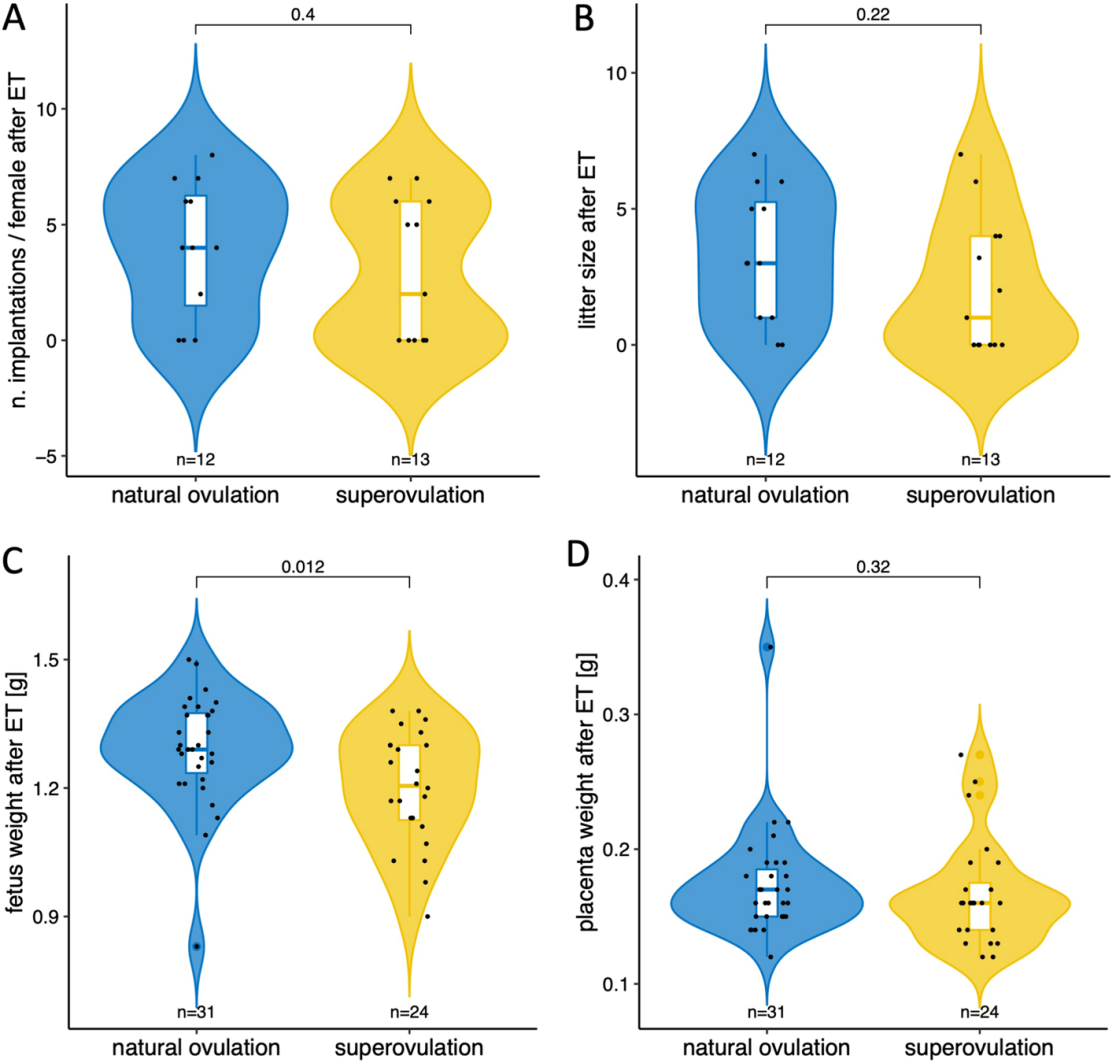
Post-implantation performance of superovulated and naturally ovulated mouse oocytes. Embryo transfers (ETs) were conducted with at least 10 recipients per group relying on fixed numbers of 4-cell stage embryos (n = 8 per pseudopregnant female). These were transplanted to the oviduct, ensuring that (i) the oocytes used were competent for cleavage, (ii) the time in culture was not unnecessarily long, (iii) the later fetal densities in the uteri were balanced between the two experimental groups, and (iv) undefined cues emanating from a stimulated genital tract were removed. (**A**) Number of implantations and (**B**) fetuses recorded at hysterectomy, 18 days after the transfer of 8 embryos to each pseudopregnant female. Each data point corresponds to one ET, i.e. one mother. P values refer to differences of numbers of implanted embryos or number of fetuses between groups (Wilcoxon test). (**C**) Birth weight and (**D**) placental weight of the individual fetuses. Each data point corresponds to one fetus or pup. P values refer to differences of weight between groups (Wilcoxon test). n, number of; ET, embryo transfer.

The mouse embryos used to study post-implantation resulted from IVC and were subjected to embryo transfer (ET; Fig. 5) to offer the same type of post-implantation environment to the two groups. Following the ET of n = 8 embryos at the 4-cell stage to pseudopregnant naturally cycling females (mated to a vasectomized male), the proportion of females that failed to become pregnant was skewed in the superovulation compared to the natural ovulation group (6 failures of 13 ETs after superovulation vs. 2 failures of 12 ETs after natural ovulation; also see the zero values for implantations and litters in Fig. 5A,B), although this skew did not achieve significance for the sample size available (p = 0.202, Fisher’s exact test). The number of fetuses recovered by hysterectomy on gestational day 18 and the number of implantations (fetuses + resorptions) were similar between the two groups (Fig. 5A,B; Supplementary Fig. S5), even when nonpregnant females were excluded from the analysis (n = 3.9 ± 2.1 vs. n = 4.0 ± 2.1 in the superovulation group *vs.* natural ovulation group, respectively). Nevertheless, the fetuses of the superovulated group had a significantly lighter body weight than those of the natural ovulation group (p = 0.012; Wilcoxon test; Fig. 5C); this was the case even if we distinguished between male and female fetuses, comparing males with males and females with females. The lighter body weight is evidently not due to the sex ratio or uterine crowding, given the fixed number of 8 embryos transplanted. It is also not a consequence of low placental weight, which was conserved in the two groups (Fig. 5D). Instead, this lighter body weight is reminiscent of the lower epiblast cell counts in the superovulated group (Fig. 4E) and consistent with the notion that the cells of the body stem from the epiblast. Apart from a lighter body weight, the pups did not present any obvious morphological anomaly.

The observations above collectively support the fact that superovulated mouse oocytes are smaller and their preimplantation embryos are developmentally inferior compared to their naturally ovulated counterparts. Embryos with a background of superovulation may also be at a disadvantage during implantation in the uterus. When implanted, however, development unfolds similarly in the two groups in terms of rates, although the fetuses of the superovulation group are lighter.

### In search of mechanisms: evidence of an insufficient duration of ovarian oocyte maturation in the etiology of the adverse superovulation effects

Two hypotheses are suitable to explain the origin of the proteome perturbation and reduced developmental fitness after superovulation. The first hypothesis considers that the exogenous gonadotropins per se made the oocyte’s proteome depart from the norm, in a direct fashion. The second hypothesis considers that the exogenous gonadotropins altered the oocyte’s normal trajectory of maturation, and this influenced the oocyte composition indirectly. In both cases, the proteome is expected to be different from that of naturally ovulated oocytes but for different reasons. Albeit, both hypotheses are not mutually exclusive; two lines of evidence favor the latter over the former.

**Figure 6:**
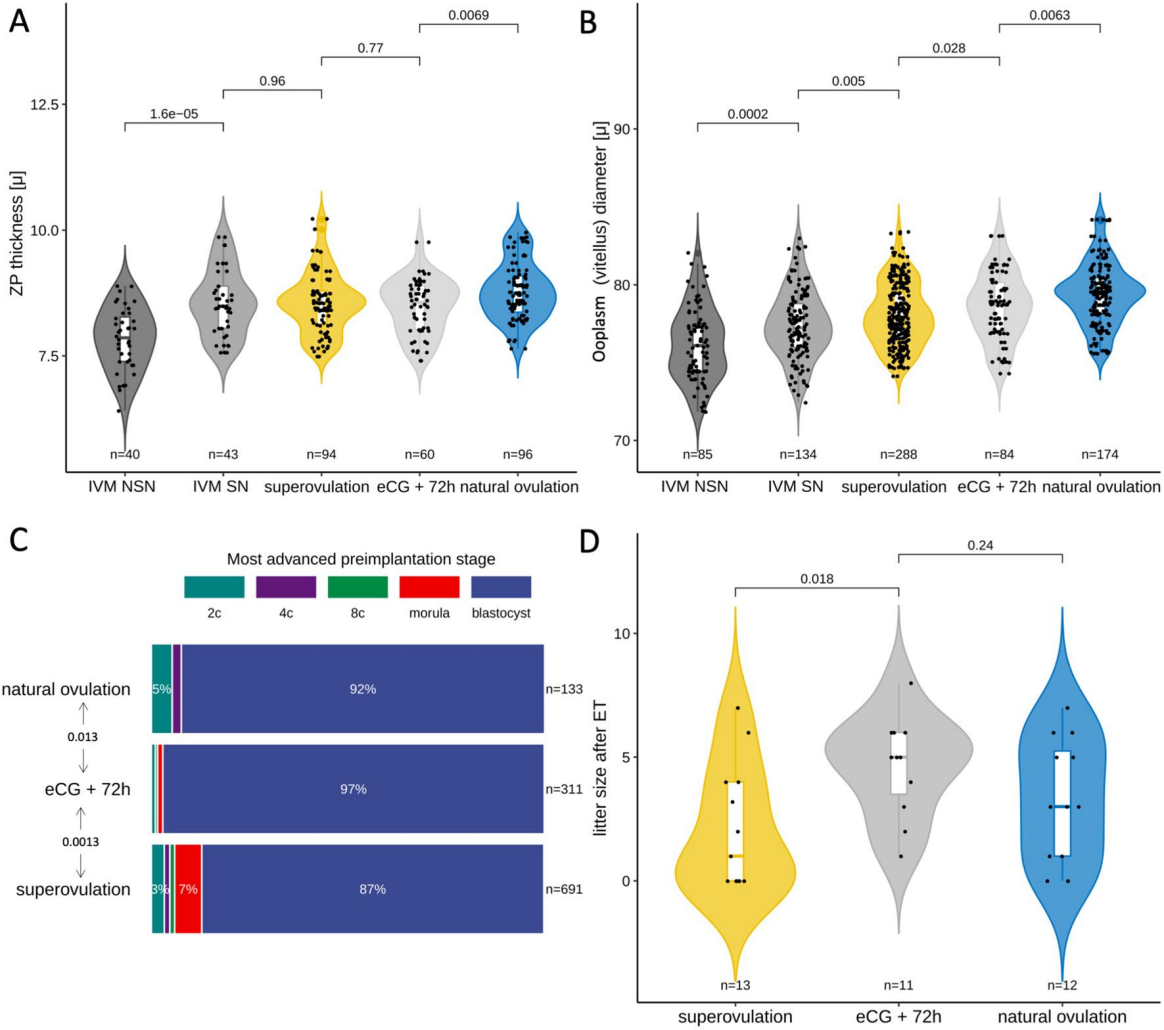
Effect of retrieving mouse oocytes before or after the standard 48 h period of ovarian maturation. (**A**) The thickness of the ZP in MII oocytes derived from germinal vesicle oocytes prior to the ovulatory stimulus of hCG (IVM), compared to oocytes ovulated after the natural stimulus or after the exogenous stimulus (48 vs. 72 h). Each data point corresponds to one oocyte. P-values refer to the differences of ZP thickness between groups (Wilcoxon test). (**B**) Diameter of oocyte proper (vitellus) in the same oocytes as described in (**A**). Each data point corresponds to one oocyte. P values refer to differences of the oocyte diameter between groups (Wilcoxon test). (**C**) The most advanced preimplantation stage reached in KSOM(aa) by the embryos of superovulated oocytes that spent 48 vs. 72 h in the ovary, compared to naturally ovulated oocytes which were cultured. Note that the scoring started from the 2-cell stage, since arrested 1-cells could be either oocytes which were not fertilized or fertilized oocytes which failed to cleave. χ^2^ test. (**D**) Number of fetuses recorded at hysterectomy, 18 days after the transfer of 8 embryos per pseudopregnant female. Each data point corresponds to one ET or mother. P-values refer to differences in the litter size between groups (Wilcoxon test). IVM, in vitro maturation; NSN, non-surrounded nucleolus; SN, surrounded nucleolus; eCG, equine chorionic gonadotropin; ZP, zona pellucida; ET, embryo transfer.

As a first line of evidence, we are going to show that the more advanced oocytes (during oogenesis) have thicker zonae. Specifically, when oocytes were matured in vitro (IVM) starting from ovarian precursors that that were known a priori to have attained a higher *vs.* lower degree of maturation, the resultant MII oocytes were distinguishable by their ZP thickness and vitellus diameter. This difference underlines the importance of the time available for oocyte maturation. In order to determine the degree of maturation of the oocytes, we took advantage of the known relationship between the extent of maturation and the chromatin distribution in the oocyte’s germinal vesicle^60^: briefly, more advanced oocytes have a germinal vesicle with a surrounded nucleolus (SN), while less mature oocytes have a non-surrounded nucleolus (NSN). Interestingly, upon IVM, the SN-derived MII oocytes narrowed the deficit of ZP thickness and cell diameter compared to NSN oocytes (Fig. 6A,B). We dissociated the two factors to facilitate interpretations since oocyte maturity and size are interdependent and they matter for development. The sole reduction of ooplasm volume by 20% via aspiration did not exacerbate the preimplantation losses (blastocyst rate 80%, n = 35; 28/35; sham manipulation, 64%, n = 26; p = 0.24, Fisher’s exact test) or the imbalance of blastocyst composition (Supplementary Fig. S6). This observation suggests that superovulation brakes the development of the oocytes before they complete maturation, and leads us to the second line of evidence.

**Figure 7:**
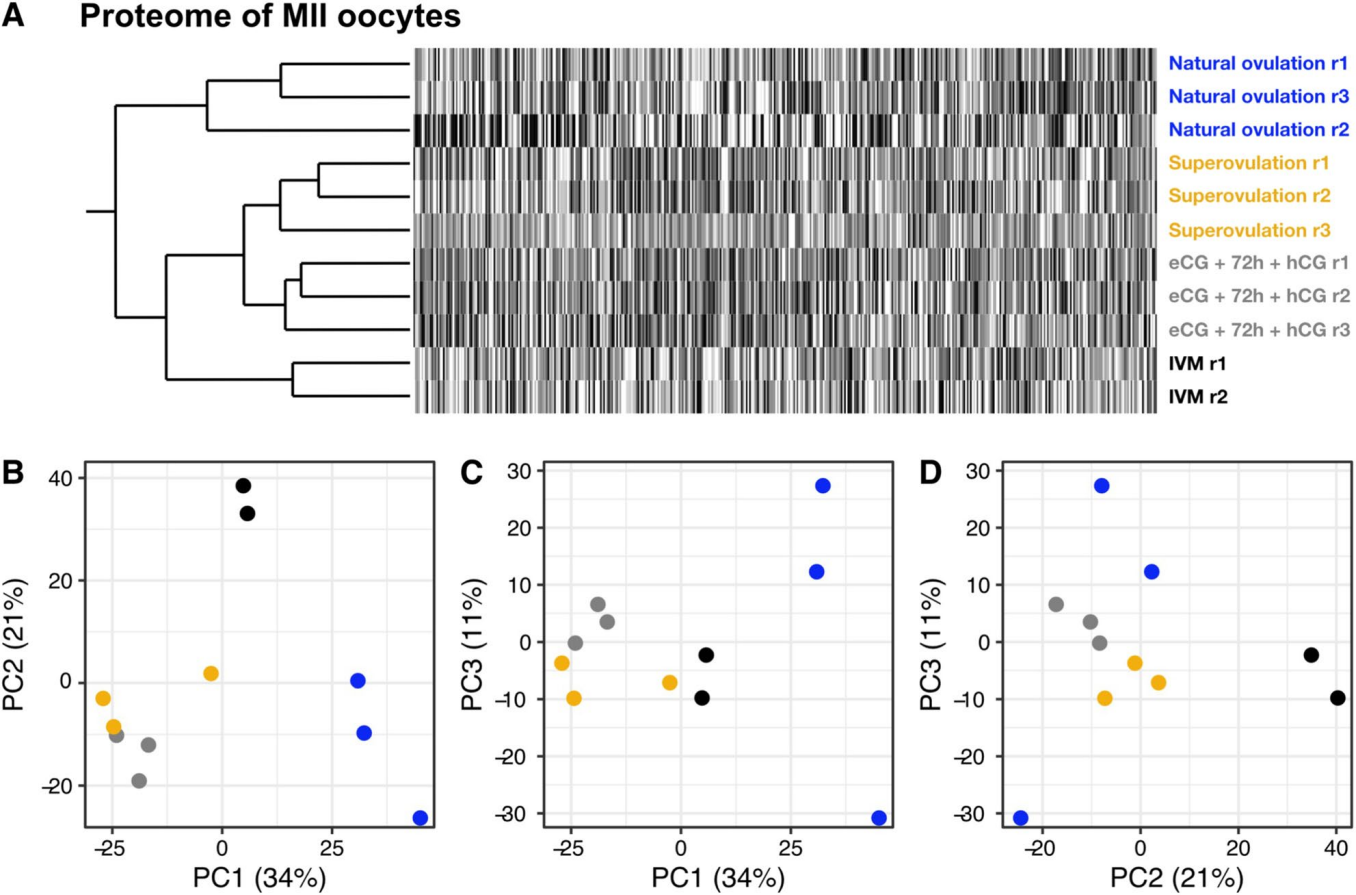
Proteome analysis (TMT) of mouse oocytes superovulated with the 72 h regime compared to conventionally superovulated, naturally ovulated or IVM oocytes. Hierarchical clustering of the proteome (**A**) and PCA (**B**–**D**). Each data point corresponds to one replicate. Oocytes superovulated with the 72 h regime form a separate subcluster (**A**). The 1st component of the PCA resolves the naturally ovulated oocytes from all the others (**B**). The 2nd component of the PCA resolves the IVM oocytes from all the others (**B**, **D**). The 3rd component of the PCA almost resolves the superovulated of the conventional stimulation protocol from the superovulated oocytes of the eCG + 72 h + hCG protocol (**C**, **D**). Samples were derived from pools of 200–250 oocytes in triplicate for each treatment (except IVM, in duplicate). IVM, in vitro maturation; eCG, equine chorionic gonadotropin; hCG, human chorionic gonadotropin; TMT, tandem mass tag.

As a second line of evidence, we will show that a shorter *vs.* longer time in maturation results in a change of oocyte’s proteome composition. Specifically, the proteomic differences between superovulated and naturally ovulated oocytes were remodeled when the time interval between the administration of the two gonadotropins was increased from 48 to 72 h. An important prerequisite for the 48–72 h extension was the knowledge that the half-life of eCG in vivo is up to 5–6 days^61,62^, which includes the 72 h. The extension of the time interval did not have any evident consequences for the number of oocytes found in the oviduct after hCG (26.9 ± 10.3 and 24.4 ± 13.2 after 48 and 72 h, respectively; total oocytes = 538 and 414, respectively; p = 0.53, Wilcoxon test). However, the increase from 48 to 72 h resulted in a splitting of the superovulated proteomes (tandem mass tag: TMT) in two groups, as revealed by hierarchical clustering (Fig. 7A) and PCA (Fig. 7B; Supplementary Table S7). In particular, PCA separated the superovulated oocytes of 48 h and the superovulated oocytes of 72 h in the 3^rd^ component (PC3, ~ 11% of the variance, Fig. 7C,D), which is the largest effect after that of superovulation compared to natural ovulation (1^st^ component, PC1, ~ 34% of the variance, Fig. 7B,C) and the effect of IVM (2^nd^ component, PC2, ~ 21% of the variance, Fig. 7B, D).

Based on these proteomic differences, we reexamined the morphological and functional parameters previously found to differ between superovulated and naturally ovulated oocytes. Morphologically, the diameters of the oocytes of the 72 h regime were closer to those of the natural ovulation group (median 78.8 vs. 79.5 μm) than the diameters of the oocytes matured in 48 h (median 77.9 μm; p ≤ 0.028), whereas no changes of ZP thickness could be reliably measured in the time from 48–72 h (Fig. 6A,B). During embryonic development in vitro (IVC)*,* the embryos derived from the oocytes of the 72 h regime were almost never arrested prior to the blastocyst stage, thereby, resembling their natural ovulation counterparts (p = 0.0013, χ^2^ test; Fig. 6C). After the ET to pseudopregnant recipients, the litter sizes of oocytes matured in 72 h were indistinguishable from those of the natural ovulation and superior to those of the 48 h superovulation (Fig. 6D). As the newborns were scored for overall normalcy, those of the 72 h regime did not present any obvious anomalies, and their body weight was similar to that of the natural ovulation group (1.25 ± 0.14 vs. 1.29 ± 0.13 g; n = 48 and 31, respectively; p = 0.056). Hence, extending the ovarian residency appears to alleviate the quality problem posed by the superovulation. This consideration is consistent with the previous results, in which volume change alone proved insufficient to alter the developmental quality (Supplementary Fig. S6).

The evidence above collectively indicates that superovulated mouse oocytes share characteristics with oocytes that are recruited for ovulation before their maturation is completed. This is consistent with these deficits also appearing in immature oocytes induced to mature in vitro and with the correction of the deficits upon an increase of the time between eCG and hCG.

## Discussion

It is commonly held that mouse oocytes ripened via superovulation possess developmental abilities that are very similar to those obtained after natural ovulation, and that residual differences are due to unrelated causes. If it were not so, then superovulated oocytes would not be suitable to derive general conclusions about development, and assisted human reproduction could be less safe than we think. We hypothesized that proteomic data, missing to date, may provide new insights into the matter. As our study shows, while the transcriptomes of superovulated mouse oocytes and derivative embryos are very similar to their naturally ovulated counterparts, the proteomes are clearly distinguishable. Therefore, the common assumption that superovulated oocytes are equivalent to naturally ovulated oocytes is undermined. Gene enrichment analysis of the proteins expressed differentially returned functionally relevant ontology terms, such as “thin ZP,” “embryonic growth arrest,” and “absent inner cell mass proliferation,” which are prominent in the first part of the discussion. How these phenotypic traits arose in superovulated oocytes and embryos, and why they were not discovered previously, forms the second part of the discussion.

Although comparison with similar studies is one of the cornerstones of scientific analysis, we could not find any proteome studies of mouse embryos derived from naturally ovulated oocytes, this being the first one. Therefore, we fell back on the transcriptome. In one of only two transcriptomic studies eligible for comparison^45,63^, blastocysts were recovered from the stimulated genital tract and 92 genes were found perturbed (1.5-fold change, p < 0.01), but it is not clear if these perturbations were caused by oocyte quality or by the environment of the stimulated genital tract^45^. In a second study, 117 of 11,110 genes were found expressed differentially between oocytes from eCG-primed and unprimed animals^63^, but the oocytes were at the germinal vesicle stage and, thus, were not directly relevant for reproduction^63^. Apart from these genome-wide studies, the effects of superovulation on mouse oocytes were studied by candidate gene approaches using qPCR and/or immunofluorescence. One study reported a significant reduction of maternal effect gene expression (Bmp15, Hdgf, Dnmt1, Dppa3, and Zfp57;^22^), another study reported the partial reduction of the amount of DNA methyltransferase protein^24^, and a third study reported altered levels of Epab and Pabpc1^42^. Our datasets (this study) do not show significant changes in these gene products, suggesting that these changes came about under laboratory-specific conditions.

Examining our proteomic dataset on its own, a remarkable finding was that the mouse oocytes and embryos, which had been manually deprived of ZP, featured just the ZP proteins among the proteins of the superovulation vs. natural ovulation expressed differentially. How could the ZP proteins be differentially expressed in oocytes which were deprived of the ZP, and in derivative embryos? Previous reports documented the presence of ZPs inside the cell, for example, in the cytoplasm and on the plasma membrane of mouse oocytes^54,64–66^. Our Western blot analysis confirmed that the bulk of Zp3 is intracellular, since the protein band had almost the same intensity in the ZP-free and -enclosed samples. We wondered if the different intracellular ZP levels measured by proteomics related to the thickness of the ZP before this was removed manually. Indeed, we verified that the ZP was thicker in the naturally ovulated than in the superovulated oocytes. This is reminiscent of the variable ZP thickness reported in human oocytes after IVM, in which, in addition to the ZP, the oocyte proper (vitellus) was also found to vary in size^67,68^. Therefore, we checked whether the superovulated mouse oocytes with thinner or thicker ZP also presented different diameters, which proved to be the case. These morphometric findings contrast with the prevalent view that superovulated and naturally ovulated oocytes have similar diameters. We conjecture that the difference has been overlooked until now due to limited sample sizes^27^, reference to unpublished data (e.g. within^69^) or the use of data obtained from non-conventional protocol of superovulation^70^. We propose that ‘small oocyte’ could be added to the MP Browser of the Mouse Genome Informatics (http://www.informatics.jax.org/vocab/mp_ontology) as a sub-term of the “regulation of cell size” (GO:0008361).

Another remarkable finding of our study was that the superovulated oocytes also gave rise to embryos with more frequent—and later occurring—arrest at preblastocyst stages; and that these blastocysts contained an inner cell mass depleted of primitive endoderm and epiblast, supporting the MP terms “embryonic growth arrest” and “absent inner cell mass proliferation.” One would expect that properly functioning embryos discard their unfit members as soon as possible, which is what the naturally ovulated oocytes did, already at the 2-cell stage; by contrast, superovulated oocytes were selected later, at the morula stage. We cannot explain the mechanism underlying the delay, but we note the chronological proximity between the morula stage and the stage when the proteome is most perturbed (8-cell stage; this study), in accord with our previous work^47^. This chronological proximity supports the idea that the later developmental arrest of superovulated oocytes was a consequence of a disturbance of protein expression. At the blastocyst stage, we recorded a depletion of the inner cell mass under the same gonadotropin dosage (10 I.U.) as used in another study^15^, although the mouse strains were different. The depletion of epiblast and primitive endoderm cells echoes our previous work^71^, in which we reported on a small – but not negligible – proportion of non-manipulated, in vivo-produced blastocysts lacking epiblast cells in their inner cell masses. This phenotype is consistent with relevant proteins returned by our GO analysis, namely, Dab2 and Cops5 (Supplementary Table S6), which are *bona fide* markers of the primitive endoderm and epiblast sub-compartment of the inner cell mass and whose genetic mutants feature defects of cell proliferation^72–77^. In contrast to the inner cell mass and its sub-compartments, the trophectoderm cell numbers were the same in both groups, in line with a report that embryo differentiation, particularly in trophectoderm cells, is influenced by embryo culture (identical for the two groups in our study) rather than by ovarian stimulation^52^.

In the second part of the discussion we would like to address the etiology of the differential protein expression associated with the superovulation (“thin ZP,” “embryonic growth arrest,” “absent inner cell mass proliferation”) and its implications. Specifically, did the mouse oocytes ‘shrink’ after the superovulation, consistent with the acquisition of cell volume regulation capacity^93^, or did the oocytes fail to attain full size by the time of the stimulus with hCG? Furthermore, did the embryos have reduced fitness because the oocytes were smaller, or was the smaller size an epiphenomenon? We, firstly, tested whether making the oocyte volume even smaller (-20%) would exacerbate the problem. The volume-reduced oocytes formed blastocysts at the same rate as sham-manipulated controls, and their germ layer composition was unaffected. Even though a 20% reduction cannot possibly change the protein abundances by, for example, the > twofold encountered in this study (in the case of superovulation), we deem it safe enough to conclude that the reduced volume is a mere epiphenomenon, which does not, per se, cause the inferiority of superovulated oocytes. Instead, we propose a scenario whereby the superovulation treatment stops the oocytes prematurely on their way to attain their proper size and natural quality, in line with what other investigators proposed based on DNA methylation data^29^. This scenario is supported by an often ignored discrepancy between the length of the natural ovarian cycle in F1 mice, which is 4 days^78^, and interval eCG-hCG, which spans only about 48 h. If the superovulated oocytes failed to attain the proper size because the 48 h were insufficient, then the logical follow-up experiment is to allow for more time until the release of the oocytes from the ovary. While a longer eCG-hCG interval had been tested before, it reached up to 60 h with conflicting results^40,41^. We, therefore, increased the interval between eCG and hCG to 72 h – time that still supports ovulation in *Calomys*^79^ and mice^80^ without incurring preovulatory aging^81^. In global terms of proteome composition, the oocytes released from the ovary after 72 h were distinguishable from the conventionally superovulated oocytes, although the differences were small at the level of the individual proteins. Echoing Pan et al.^63^, our interpretation of the widespread but small differences is that “egg quality is unlikely the outcome of expression of a highly restricted number of genes”^63^. Phenotypically, the oocytes released from the ovary after 72 h had a diameter which was closer to that of naturally ovulated oocytes. The similarity of the two groups also emerged from the occurrence of preblastocyst arrest, which almost never occurred in the 72 h protocol. Post-implantation development also seemed to benefit from the extension, whereby the litter size after 72 h was no longer inferior to that of the natural ovulation. These improvements after 72 h support the fact that the oocytes had stopped prematurely on their way to attain the proper size and quality in the conventional superovulation regime. It may be noted that a longer stimulation can also be beneficial in human reproduction^82^.

In spite of the results of the preimplantation phase and its superovulation-sensitive gene ontology terms (“thin ZP,” “embryonic growth arrest,” and “absent inner cell mass proliferation”), no deficits were observed following ET in females cycling naturally. Specifically, there were no significant differences in the ability to implant, and no morphological anomalies were apparent in the fetuses of superovulated ovulated oocytes, except for a slight reduction of body weight, compared to the naturally ovulated group. Although more experiments, more mice and more embryos would probably increase the statistical power and expose additional and more subtle effects of the superovulation, there should be an ethical balance between the hypothetical gain of knowledge and the consumption of animals. It seems questionable to increase the number of experiments until statistical significance is reached^83^ if conclusions can already be drawn without killing extra animals. The slight reduction of body weight is consistent with an independent mouse study^38^, while it seems to be at variance with the conclusion of another independent study reporting that “preimplantation embryo exposure to superovulation affects placental growth, whereas peri-implantation exposure affects fetal growth”^39^. One experimental difference between the studies is that zygotic cleavage took place in vitro in our study, whereas in the other study, blastocysts were flushed from the uterus^39^. The lack of obvious fetal anomalies is also in line with our previous study^84^, in which we reported that the preimplantation effects of embryo culture media do not persist after implantation. It may be noted that a background of hormonal stimulation was also associated with reduced birth weight with human oocytes, and this reduction was observed no matter whether the uterus had been stimulated or not^85,86^. Although our current data do not rule out more subtle or long-term differences between superovulated and naturally ovulated oocytes, they support a scenario of negative selection of the unfit or unviable embryos as occurring during preimplantation, whereby there remains less, if anything, left for later selection.

In summary, our tandem transcriptome and proteome analysis revealed that the superovulation is not inflicting direct injury on the mouse oocytes, but causes them to be released from the ovary prematurely, before their proteomes reach a state equivalent to that of natural ovulation. Our proposal allows the reconciliation of the different views on superovulation: the view that superovulated oocytes are identical to naturally ovulated ones (with differences being due to confounding factors, for example), and the view that superovulated oocytes are inherently different from naturally ovulated ones (as documented for specific genes). Both views are correct, depending on the gene expression product, whether transcript or protein, and on the time elapsed until superovulation. Since oocyte composition and response to gonadotropins vary between mouse strains^6,49^, the magnitude of the superovulation effect cannot be extrapolated from this study to other studies, but it needs to be measured case by case. Regardless of the effect size, we are convinced that there is more to be found in the developmental proteome than in the transcriptome. We propose that proteome analysis bring benefits to a modern understanding of oocyte quality: not only is the proteome closer to the phenotype, but the protein information cannot be reliably inferred from transcriptome studies. If we continue to rely on transcripts in lieu of transcripts plus proteins, our molecular understanding of the oocyte quality will stay woefully incomplete. If we persist in using the routine 48 h protocol which prevents superovulated mouse oocytes from attaining their proper size, the oocyte quality itself may stay lower than necessary. Unfortunately, the current protocol for superovulation reduces the number of mice for oocyte production only to increase the number of mice for embryo production, which means, no net difference and no reduction of animal usage in the sense of the 3 Rs^7^. Ultimately, an evidence-based protocol for superovulation may be quite different from the one currently used routinely in mice, and also the ovarian stimulation widely used in assisted human reproduction could be less safe than we think.

## Materials and methods

### Compliance with regulations on research animals

All mice used in this study (N = 1067 females, 240 males over a period of approximately 5 years) were reared in-house at the MPI Münster. They were maintained in individually ventilated type 2 L cages (Ehret), with autoclaved Aspen wood as bedding material and a cardboard tube as enrichment, in groups of 5 females or individually as males. Access to water (acidified to pH 2.5) and food (Teklad 2020SX, Envigo) was ad libitum. The animal room was maintained at a controlled temperature of 22 °C, a relative humidity of 55%, and a 14/10 h light/dark photoperiod (light on at 6:00 a.m.). The hygiene status was monitored every three months as recommended by Federation of European Laboratory Animal Science Associations (FELASA), and the sentinel mice were found free of most pathogens except mouse norovirus, *Helicobacter spp.*, *Pasteurella spp.*, and *Trichomonas spp.*. Procedures used in this study followed the ethical guidelines of the FELASA and the ARRIVE reporting guidelines^87,88^. On the regulatory level, mice were used for experiments in compliance with the European, national and institutional guidelines and approved by the Landesamt für Natur, Umwelt und Verbraucherschutz (LANUV) of the state of North RhineWestphalia, Germany (Permit number 84-02.04.2016.A229; 81-02.04.2017.A432).

### Gonadotropin stimulation regime

Equine chorionic gonadotropin (eCG), also known as pregnant mare serum gonadotropin, and human chorionic gonadotropin (hCG) were obtained from Intervet as commercial products Intergonan and Ovogest, respectively (MSD Tiergesundheit, Germany). Lean B6C3F1 mice aged 8–10 weeks and weighing approx. 25 g were injected intraperitoneally, using a 27G needle, at 5 pm, with 10 I.U. eCG and 10 I.U. hCG 48 h apart, as per the conventional superovulation protocol in use since 1957^4^. Ten I.U. corresponds to 128 ng eCG^62^ and compares to the 335 ng/mL FSH found in the plasma of C57Bl/6 mice^89^. The interval between eCG and hCG was increased to 72 h in one set of experiments.

### Collection and morphometry of unfertilized and fertilized oocytes from natural and stimulated cycles

Regarding natural ovulation, 8–10-week-old B6C3F1 females were injected with a sole vehicle used to dissolve the gonadotropins (PBS) and caged three days with vasectomized or stud CD1 males (6–12 months old), as mating trios (2 females, 1 male). A vaginal plug was taken as indicating that mating had occurred. At 8 a.m. on the third day after caging, the females plugged on that day were killed by cervical dislocation and metaphase II (MII) oocytes or fertilized oocytes (1-cell embryos) were isolated. Regarding superovulation, B6C3F1 females were injected with eCG and hCG 48 or 72 h apart at 5 p.m., and upon the second injection, were caged as mating pairs (1 female, 1 male) for one night with vasectomized or stud CD1 males. On the following day at 8 a.m., the plugged females were killed and the oocytes isolated as follows. The cumulus-oocyte complexes were collected by tearing the oviducts and released into Hepes-buffered Chatot, Ziomek and Bavister medium (HCZB)^90^ with bovine serum albumin (BSA) replaced by polyvinylpyrrolidone (40 kDa). The cumulus-oocyte complexes were brought to the laboratory, where further processing took place at 28 °C room temperature (RT). Cumulus cells were removed in hyaluronidase (cat. no. *151271*, ICN Biomedicals, USA; 50 I.U./mL in HCZB) on the stage of a stereomicroscope fitted with a camera. We were very careful to measure the oocyte proper (vitellus) rapidly and synchronously for the two groups, being aware that oocyte volume is not a constant but is dynamically regulated^91–93^. The time between the initiation of the hyaluronidase treatment and imaging did not exceed 20 min. Images were processed using NIH ImageJ software, manually drawing a line across the ZP perpendicular to its surface and measuring the zona thickness directly; the oocyte perimeter was drawn along the oolemma with a free-hand tool. The MII oocytes from natural ovulation and superovulation were transferred to fresh HCZB on the stage of an inverted microscope, and imaged side by side using a 10X objective. Measurements were converted from pixels to μm with a calibrated micrometer slide. The oocyte diameter was obtained by dividing the perimeter by pi (~ 3.141).

### In vitro production of MII oocytes from germinal vesicle oocytes

In order to obtain MII oocytes from germinal vesicle-stage oocytes via in vitro maturation (IVM), germinal vesicle-stage oocytes were retrieved from the ovary 48 h after eCG by puncturing its surface in HCZB. Oocytes were deprived of surrounding cumulus cells by gentle pipetting and incubated for 20 min at 37 °C in HCZB with the addition of 0.05 μg/mL Hoechst. Fluorescent imaging was performed on the stage of an inverted microscope using a 20X S-Fluor objective. The oocytes were sorted into two groups according to the distribution of chromatin around the nucleolus (SN or NSN;^60^) and allowed to mature overnight in α-MEM (cat. no. M4526, Sigma-Aldrich Chemie GmbH, Germany) supplemented with 10% fetal bovine serum and 50 I.U./mL gentamicin sulfate (MP Biomedicals). The SN-MII and NSN-MII oocytes were imaged side by side using a 10X objective, as described for the (super)ovulated oocytes.

### In vitro embryo culture (IVC) for proteome, transcriptome and phenotypic analysis

Pools of approximately 100 MII or fertilized oocytes were transferred to 500 μL of potassium (K) simplex optimization medium enriched with aminoacids (KSOM(aa)) in a 4-well plate without oil overlay, at 37 °C under 6% CO_2_ in air. The KSOM (aa) was synthesized in-house from individual components and included 0.5X EAA, 0.5X NEAA and 0.5X glutamine according to the recipe^94^. An amount of 0.2% (w/v) BSA (Probumin, Serologicals Corporation, Celliance) and 50 mg/L gentamicin sulfate (MP Biomedicals) were added to the KSOM(aa). The medium was not changed during embryo culture, but the embryos were moved to another well filled with medium 48 h earlier. Developmental stages were collected from the plate at the desired time points (MII oocyte, 15 h post hCG (hphCG); 1-cell stage, 15 hphCG; 2-cell stage, 43 hphCG; 4-cell stage: 53 hphCG; 8-cell stage, 62 hphCG; morula: 72 hphCG; blastocyst, 92 hphCG; the non-stimulated counterparts were collected at the same time as the superovulated oocytes/embryos). Regarding proteome analysis, oocytes and embryos were deprived of the ZP by pipetting in warm acidic Tyrode solution (cat. no. T1788, Sigma-Aldrich Chemie GmbH, Germany) for 30–60 s and then rinsed in protein-free Hepes-buffered CZB medium (BSA replaced through polyvinylpyrrolidone 40 kDa). About 200–250 oocytes or embryos were lysed in 15–20 μL of sodium dodecyl sulfate (SDS) lysis buffer (4% SDS, 50 mM HEPES pH 7.5) to produce each individual sample and stored at − 80 °C until further processing. Regarding transcriptome analysis, an average of 210 oocytes or embryos per sample were lysed, still encased in their zona, in lysis buffer (Zymo Research, cat.no. R1051) and stored at − 80 °C until further processing. The developmental stages were also inspected for the occurrence of cleavage arrest, and sampled for ETs and the assessment of the blastocyst’s cell lineage allocation.

### Proteome analysis of MII oocytes, pronuclear oocytes and preimplantation embryos

The following methods essentially follow those of our previous work^46^. In order to compare the developmental series between superovulation and natural ovulation, we made use of our established analysis pipeline (^51^, PXD003093;^50^, PXD000512), which is based on the ‘stable isotope labeling by/with amino acids in cell culture’ (SILAC) technique^95^. In addition, we used a multiplexing pipeline with TMT^53^, to compare the sole MII oocytes between superovulation and natural ovulation.

Samples were spiked with a standard prepared from F9 embryonal carcinoma (EC) cells for SILAC^96,97^. F9 EC cells build tumors (teratomata) that are considered as caricatures of embryogenesis, because they can differentiate into almost every tissue^98^, therefore, F9 EC cells afford an ample coverage of the proteins expressed in early embryos. Each oocyte or embryo lysate was supplemented with an equal amount of protein lysate from isotopically labeled (Lys8 and Arg10) F9 EC cells as SILAC spike-in standard (> 98% labeling efficiency). These 1:1 mixtures were then digested with Lysyl endopeptidase and trypsin, desalted, and fractionated by offline high-pH reversed-phase chromatography. Fractionation was accomplished in a concatenated fashion. Lastly, all samples were analyzed by LC–MS/MS using a QExactive or QExactive HF mass spectrometer, as described in our previous work. Data were obtained in duplicate, and raw data were processed for identification and quantification by MaxQuant Software (version 1.6.4.0^99^), with the ‘iBAQ’ option enabled and the ‘requantify’ option disabled. The search for identification was performed against the UniProt mouse database (release identifier UP000000589_10090.fasta) concatenated with reversed sequence versions of all entries and supplemented with common lab contaminants. Parameters defined for the search were trypsin as the digesting enzyme, allowing two missed cleavages, a minimum length of six amino acids, carbamidomethylation at cysteine residues as a fixed modification, oxidation at methionine, and protein N-terminal acetylation as variable modifications. The maximum mass deviation allowed was 20 ppm for the MS and 0.5 Da for the MS/MS scans. Protein groups were regarded as identified with a false discovery rate (FDR) set to 1% for all peptide and protein identifications; in addition, at least two matching peptides were required and at least one of these peptides had to be unique to the protein group. We only focused on the iBAQ values of the ‘light’ peptide versions (= peptides derived from oocyte proteins but not from the F9 spike-in standard). The iBAQ algorithm allows one to calculate the abundance of proteins within one sample by summing all peptide peak intensities detected for a given protein and normalizing it by the number of theoretically observable tryptic peptides for this protein. Thus, a mass-related measure (intensity) is transformed into a measure that is proportional to molar amounts (iBAQ). The iBAQ values for each protein were then divided by the sum of all the *n* iBAQ values for a given experiment to determine the molar fractional content of each protein P (riBAQ_P_) in a sample, according to Shin et al.^100^, as follows:

$${\text{riBAQ}} = \frac{{{\text{iBAQ}}_{{\text{i}}} }}{{\sum\nolimits_{{{\text{i}} = 1}}^{{\text{n}}} {{\text{iBAQ}}_{{\text{i}}} } }}$$

We applied nonparametric normalization, which is robust to outliers and anomalies in data that were generated in distinct LC–MS/MS runs. Proteins with riBAQ_P_ values greater than zero in fewer than two samples were discarded from further analysis. The remaining riBAQ_P_ values were averaged across replicates (if the protein had been detected in both replicates; otherwise, we used the single value detected), quantile-normalized, and log_10_ transformed. These values are further referred to as “expression values” of the proteins.

Regarding TMT aimed at comparing the sole MII oocytes, we performed an 11-plex isobaric labelling experiment (isobaric reagents cat. no. 90110 and cat.no. A37724, Thermo Fisher Scientific, Germany) involving oocyte pools obtained by natural ovulation (n = 803), superovulation with a conventional 48 h interval between eCG and hCG (n = 822), and superovulation with an interval between eCG and hCG increased to 72 h (n = 666). Each of the three pools of MII oocytes was divided equally into three technical replicates. We also included single samples of IVM SN oocytes (n = 351) and IVM NSN oocytes (n = 244), which served as outgroup samples to control for similarities/dissimilarities in any subsequent dimensionality reduction analysis, such as PCA. All samples were predigested with 0.5 µg endoproteinase LysC for 2 h at 37 °C, followed by dilution with 50 mM ammonium bicarbonate buffer to lower the guanidinium hydrochloride concentration and a continuation of the digest overnight after the addition of 0.5 µg trypsin. Digestion mixtures were quenched by the addition of trifluoroacetic acid to a final concentration of 1% and desalted using C18 Stage tips. After drying samples in an Eppendorf Concentrator, peptides were resuspended in 20 µL 200 mM ethylpiperazinepropanesulfonic acid and labelled with TMT using 5 µL of each labeling reagent (about 100 µg). Subsequent to mixing at equal ratios, TMT-labeled samples were fractionated offline by high pH reversed phase HPLC on a YMC C18 column (2.1 × 100 mm; Buffer A 10 mM NH_4_OH; Buffer B 10 mM NH_4_OH 90% acetonitrile; linear gradient from 3 to 40% B in 53 min, flow rate 0.3 mL/min). Fifteen concatenated fractions were collected, lyophilized and subjected to LC–MS/MS on a Q Exactive HF instrument using a top 15 data-dependent method (scan range 350–1600 m/z; MS1 resolution 120,000; AGC target 3e6; maximum IT 50 ms; MS2 resolution 60,000; AGC target 1e5; maximum IT 108 ms; normalized collision energy = 32 V; dynamic exclusion enabled for 30 s). Raw data were processed for identification and MS2-based quantification of reporter ions by MaxQuant Software (version 1.6.17.0,^99^). The search for identification was performed against the UniProt mouse database (UP000000589_10090.fasta; release 04/2019) concatenated with reversed sequence versions of all entries and supplemented with common contaminants. Parameters defined for the search were trypsin as the digesting enzyme, allowing two missed cleavages, a minimum length of six amino acids, carbamidomethylation at cysteine residues as a fixed modification, and oxidation at methionine and protein N-terminal acetylation as variable modifications. The maximum mass deviation allowed was 20 ppm for the MS and 0.5 Da for the MS/MS scans. Protein groups were regarded as identified with an FDR set to 1% for all peptide and protein identifications; in addition, at least two matching peptides were required and at least one of these peptides had to be unique to the protein group.

### Western blot confirmation of Zp3 protein as abundantly present in zona-free oocytes

The following methods essentially follow those of our previous work^101^. Zona-free oocytes were centrifuged in protein-free HCZB medium at 700 rpm for 20 min to form a tiny pellet. The supernatant was carefully aspirated using a mouth-operated micropipette and replaced by RIPA buffer containing protease inhibitors. The resultant lysates were mixed with 6 × Laemmli sample buffer and boiled for 5 min at 99 °C. These samples were loaded on a 12% polyacrylamide separation gel blotted onto a polyvinylidene fluoride membrane. The membrane was blocked for at least 3 h and incubated (3% nonfat dry milk in 0.1% PBS-Tween) with rabbit anti-ZP3 primary antibody (cat.no. PA5-89033, Thermo Fisher Scientific, Germany) applied at a dilution factor of 1:20,000, overnight at 4 °C. After 3X washing in 0.1% PBS-Tween, the blot was incubated with horseradish peroxidase-coupled secondary antibody at RT for 1 h. The membrane was washed and then developed with chemiluminescent horseradish peroxidase substrate solution. The chemiluminescent signal was detected using the AGFA Curix 60. Signal intensities were standardized on α-Tubulin, whose antibody (cat. no. T6199, Sigma-Aldrich Chemie GmbH, Germany) was applied at a dilution factor of 1:5000.

### Transcriptome analysis of MII oocytes, pronuclear oocytes and preimplantation embryos

Total RNA was converted to cDNA using the Smarter system (Takara) and sequencing libraries were prepared using the Nextera kit (Illumina). Libraries were sequenced on an Illumina HiSeq3000 platform to obtain ~ 43 million 36-base-single-end reads per library. Raw sequencing reads were trimmed, mapped to the Mus musculus Ensembl GRCm38 assembly, and quantified as previously described^47^. Only Ensembl gene identifiers for protein-coding genes associated with one or more reads were considered for further analysis. A matrix containing the number of reads mapped to each of the remaining Ensembl gene identifiers for each sample was used as input for the DESeq2 R/Bioconductor package (version 1.24.0^102,103^), and normalized for library size with its default method. Regularized log-transformed values were obtained from the normalized counts, by applying DESeq2's rlogTransformation function with the “blind” parameter set to “TRUE” (i.e. free of experimental design). Regularized log-transformed values for Ensembl gene identifiers corresponding to the same gene symbol were averaged, and the values associated with each gene symbol were averaged across replicates. The resulting values are further referred to as “expression values” of the transcripts.

### Differential gene expression analysis

Expression values for each protein/transcript were compared between the superovulation and natural ovulation groups using a two-sided exact permutation test based on the Wilcoxon signed-rank test statistic. The Wilcoxon signed-rank test is a nonparametric test used to assess differences in the mean ranks of two paired samples. Expression values were paired by stage. Thus, for each protein/transcript, the seven developmental stages of the oocyte and derivative embryos in the superovulation group were compared to their natural ovulation counterparts, obtaining one P value. The resulting P values were corrected utilizing Benjamini–Hochberg’s method (false discovery rate < 0.1).

### Functional enrichment analysis

Functional enrichment analysis was performed with Enrichr^104,105^ at https://maayanlab.cloud/Enrichr/. We selected the MP ontology, which builds on the Mouse Genome Informatics database and is, therefore, well-suited to examine genes relevant to the mouse and its developmental biology. Terms with a FDR ≤ 0.01 were considered enriched.

### Data accessibility

The mass spectrometry proteomics data generated and analyzed in this study have been deposited to the ProteomeXchange Consortium via the PRIDE partner repository^106,107^ with the dataset identifier PXD021331 (SILAC) and PXD026347 (TMT). The RNAseq data analyzed in this study have been deposited to the DNA Databank of Japan Sequence Read Archive with the dataset identifier DRA005956 and DRA006335. A microarray dataset obtained from the same cellular material as the RNAseq has been deposited in the NCBI’s Gene Expression Omnibus and is accessible through GEO Series accession number GSE110599.

For convenience, easy-access data tables of PXD021331, DRA005956-DRA006335, and PXD026347 are provided in the supplementary material as Supplementary Tables S1, S2, and S7, respectively.

### Embryo transfer and post-implantation development

Groups of eight embryos were removed from culture at the 4-cell stage and transferred surgically to one oviduct of pseudopregnant CD1 recipients. Pseudopregnancy was induced by mating untreated females with vasectomized CD1 males. On the day of the copulation plug, the females weighed between 27 and 33 g and were older than 8 weeks but no older than 3 months. Prior to surgery, CD1 recipients were anesthetized with Ketamin (80 mg/kg body weight)/Xylazin (16 mg/kg)/Tramadol (15 mg/kg) in PBS, delivered intraperitoneally, as per our LANUV approval. The eyeballs were covered with eye ointment (Bepanthen) to protect the cornea. The surgical tools and the operating table were wiped with disinfectant (Sterilium). A 1-cm incision was made in the skin along the dorsal midline using disinfected scissors and tweezers, and then a 3-mm paralumbar incision was made in the peritoneum just above the fat pad, which is characteristically located close to the kidney and ovary. The ovary and ancillary tissues were gently pulled out, and kept in position using Serafin clips. Drops of epinephrine solution (0.1 mg/mL in PBS) were applied to the surface of the *bursa ovarica* to reduce bleeding when tearing the bursa to expose the oviduct and its infundibulum using fine tweezers. Embryos were deposited in the infundibulum using a mouth-operated glass pipette with a flame-polished tip. After 2–3 min to allow the embryos to settle inside the oviduct, the tissues were returned to the original position and the wound in the skin was closed with Michel Suture Clips. The surgery per se took typically 10–15 min per mouse. The post-surgery recovery area was warmed to ≈ 30 °C using infrared lamps. Animals were returned to their cages when fully awake, and were given postoperative analgesia via Tramadol in the drinking water (1 mg/mL) for 3 days, as per our LANUV approval. CD1 females were killed by cervical dislocation 18 days post-ET at E18.5, and then subjected to hysterectomy. The number of uterine implantation sites and resorption sites were counted for each animal. Fetuses and placentae were weighed and inspected for signs of obvious growth retardation and obvious external malformations, if any.

### Experimental volume reduction of the ooplasm

Pronuclear-stage oocytes were incubated in 5 μM Latrunculin B (dilution of 1000X stock in DMSO; cat. no. 428020, Merck Millipore, Darmstadt, Germany) diluted in Hepes-buffered CZB. About 30–35 picoliters of ooplasm were aspirated using a TransferTip (ES) pipette (cat. no. 5195000079, Eppendorf) operated by a CellTram Vario (Eppendorf). Manipulated oocytes were washed and returned to culture in KSOM(aa) for further development.

### Analysis of cell lineage allocation of blastocysts

Blastocysts from superovulated or naturally ovulated oocytes were analyzed by performing an immunostaining followed by confocal microscopy imaging to identify and map the different cell lineages, as described^72,108^. The following primary antibodies were applied simultaneously to the specimens overnight at 4 °C: anti-Cdx2 mouse IgG1κ (cat. no. CDX2–88, Emergo Europe, The Hague, The Netherlands), anti-Nanog rabbit IgG (cat. no. REC-RCAB0002P-F, Cosmo Bio, Tokyo, Japan), and anti-Sox17 goat IgG (cat no. AF1924, R&D Systems) in dilutions of 1:200, 1:2000, and 1:100, respectively. Appropriate Alexa Fluor-tagged secondary antibodies (Invitrogen) were matched to the primaries and incubated for 2 h at RT. Embryos were placed in 5 μL drops of PBS on a 50-mm thin-bottomed plastic dish (Greiner Bio-One, Lumox hydrophilic dish; Frickenhausen, Germany) and overlaid with mineral oil (M8410 Sigma). Images were captured on the stage of an inverted microscope (Eclipse 2000-U; Nikon, Düsseldorf, Germany) fitted with a spinning disk confocal unit (Ultra View RS3; Perkin-Elmer LAS, Jügesheim, Germany). A Nikon Plan Fluor 40X oil immersion lens (NA 1.30) was used. Twenty optical sections per blastocyst were captured using a Hamamatsu ORCA ER digital camera (Hamamatsu Photonics KK, Japan). Maximum intensity projections were analyzed with ImageJ Version 1.46j, counting the CDX2-, SOX17- and NANOG-positive cells manually.

### Statistical analysis of morphometric data, gene expression data and developmental rates

The ZP thickness, diameter of oocyte proper (vitellus), fetal rates, and placental weights were analyzed by Wilcoxon using the statistical program JMP v.13 (SAS Institute Inc., USA). Developmental arrest at preimplantation stages and proportions of cell lineages in blastocysts were analyzed using the Fisher’s Exact test.

### Ethics declaration for human experiments and consent for publication

Not applicable.

### Ethics declaration for animal experiments

Mice were used for experiments according to the license issued by the Landesamt für Natur, Umwelt und Verbraucherschutz of the State of North Rhine-Westphalia, Germany (license number 81-02.04.2017.A432 and 84-02.04.2016.A229), in accordance with the procedures laid down in the European Directive 2010/63/EU. We observed the ARRIVE guidelines^87,88^ to the extent applicable, considering that the bulk of our experiments were conducted in vitro not in vivo, with the exception of ETs.

## Supplementary Information

Supplementary Information.

## Data Availability

All data underlying this article are available herein (Supplementary Tables) and in the following repositories. The raw data of Supplementary Table S1 (mass spectrometry) have been deposited to the ProteomeXchange Consortium via the PRIDE partner repository with the dataset identifier PXD021331. The raw data of Supplementary Table S2 (transcriptome analysis) have been deposited in the DNA Databank of Japan Sequence Read Archive with the dataset identifiers DRA005956 and DRA006335. An additional transcriptomic dataset is publicly available in NCBI’s Gene Expression Omnibus and is accessible through GEO Series accession number GSE110599. The raw data of Supplementary Table S7 (mass spectrometry) have been deposited to the ProteomeXchange Consortium via the PRIDE partner repository with the dataset identifier PXD026347.
