# Supplementary material for: The proteome, not the transcriptome, predicts that oocyte superovulation affects embryonic phenotypes in mice
Source: Sci Rep. 2021 Dec 9;11:23731. doi: 10.1038/s41598-021-03054-9 (PMC8660899; doi:10.1038/s41598-021-03054-9)
Supplement: Supplementary file 1 — Supplementary Information. [file 41598_2021_3054_MOESM1_ESM.pdf]

Supplementary information

The proteome, not the transcriptome, predicts that oocyte superovulation affects embryonic phenotypes in mice

Authors: Leila Taher[1,*], Steffen Israel[2], Hannes C.A. Drexler[2], Wojciech Makalowski[4], Yutaka Suzuki[5], Georg Fuellen[3,*], Michele Boiani[2,*]

[1] Graz University of Technology, Institute of Biomedical Informatics, Stremayrgasse 16/I, A-8010 Graz

[2] Max Planck Institute for Molecular Biomedicine, Roentgenstrasse 20, 48149 Muenster, Germany

[3] Rostock University Medical Center, Institute for Biostatistics and Informatics in Medicine and Aging Research (IBIMA), Ernst-Heydemann-Strasse 8, 18057 Rostock, Germany

[4] Institute of Bioinformatics, Faculty of Medicine, University of Münster, Niels Stensen Str. 14, 48149, Münster, Germany.

[5] Department of Medical Genome Sciences, Graduate School of Frontier Sciences, University of Tokyo, Kashiwa, Chiba, 277-8562, Japan.

List of supplementary items

Supplementary Figure S1: Morphologies of the oocytes and embryos used in this study, and Western blot evidence that Zp3 is intracellular.
Supplementary Figure S2: Adverse effect of undefined cues emanating from the genital tract of stimulated females.
Supplementary Figure S3: Expression analysis of the full transcriptome (n=20,875).
Supplementary Figure S4: Distribution of the expression values of the differentially expressed proteins and transcripts, compared to those of the 2,844 constitutively expressed proteins and their transcript counterparts.
Supplementary Figure S5: Representative cases of hysterectomies conducted on day 18 after embryo transfer, related to Figure 5.
Supplementary Figure S6: Oocyte size *per se* is not causative of reduced embryonic fitness.
Supplementary Figure S7: The uncropped Western blot images of Zp3 and α-tubulin shown in Supplementary Figure S1.

Supplementary Table S1: Dataset of 6444 proteins, $riBAQ_P$ values.
Supplementary Table S2: Dataset of 20875 transcripts, regularized log-transformed values obtained from normalized counts.
Supplementary Table S3: Datasets of 2844 constitutive proteins, with $riBAQ_P$ values averaged across replicates, quantile-normalized, and log10 transformed.
Supplementary Table S4: Differential expression analysis of the constitutive proteome.
Supplementary Table S5: Differential expression analysis of the transcriptome.
Supplementary Table S6: Proteins describing the MP terms “thin zona pellucida”, “embryonic growth arrest” and “absent inner cell mass proliferation”, compared to housekeeping proteins; $riBAQ_P$ values and their ranked values.
Supplementary Table S7: Dataset of 1857 proteins, TMT values.

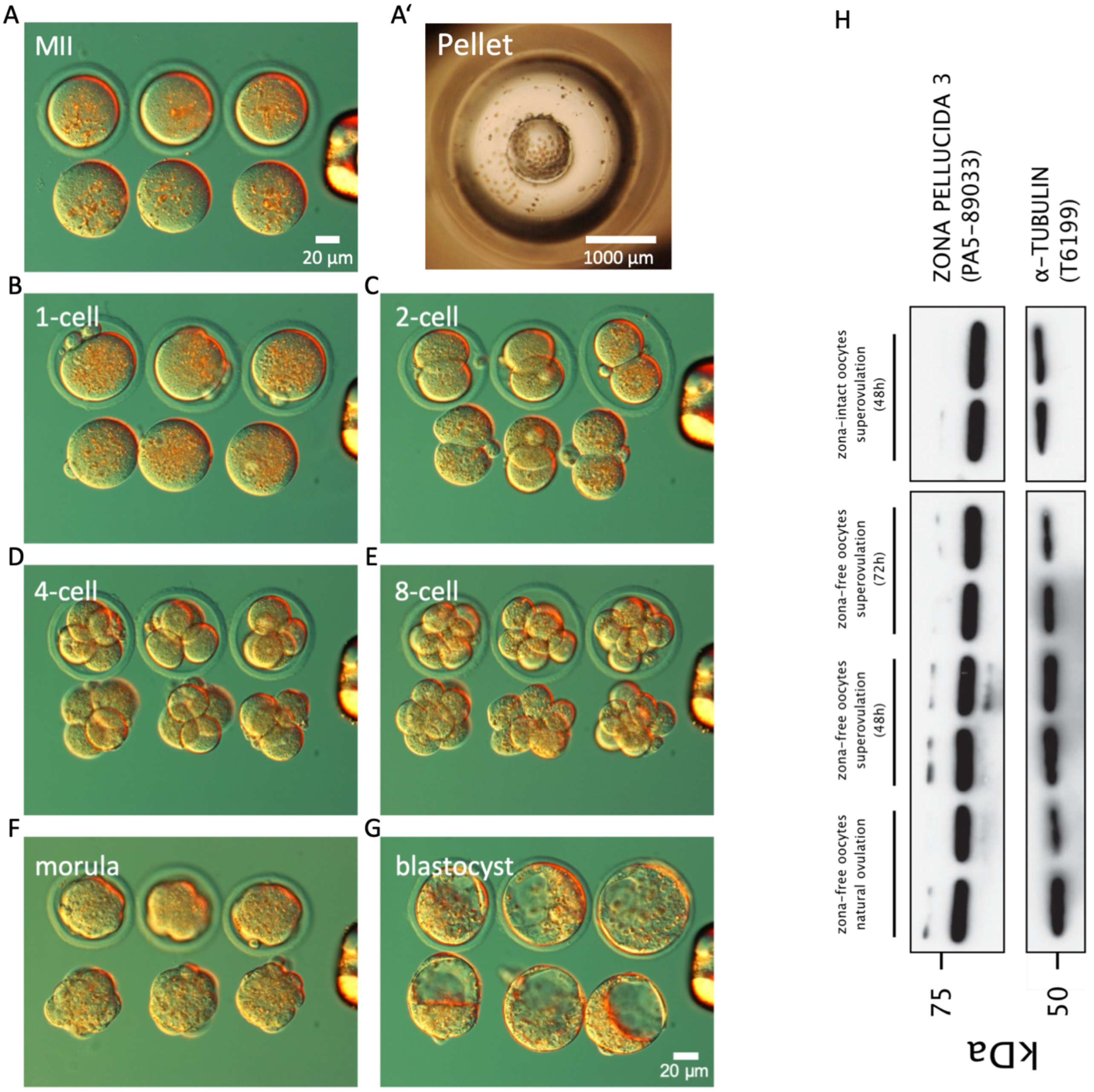

Supplementary Figure 1. Morphologies of the oocytes and embryos used in this study, and Western blot evidence that Zp3 is intracellular. Note that the zona pellucida (upper series in each high-magnification panel, A-G) was removed completely to produce the samples of the proteome analysis (lower series in each high-magnification panel, A-G). Pellet (top right, A') is a stereomicroscopic view of the zona-free oocytes collected on the bottom of a tube after centrifugation, prior to further processing for mass spectrometry or Western blotting. H. Western blot image showing that Zp3 is abundantly present in MII oocytes irrespective of whether the zona was removed (zona-free) or was left in place (zona-intact). The uncropped blots are presented in Supplementary Figure S7.

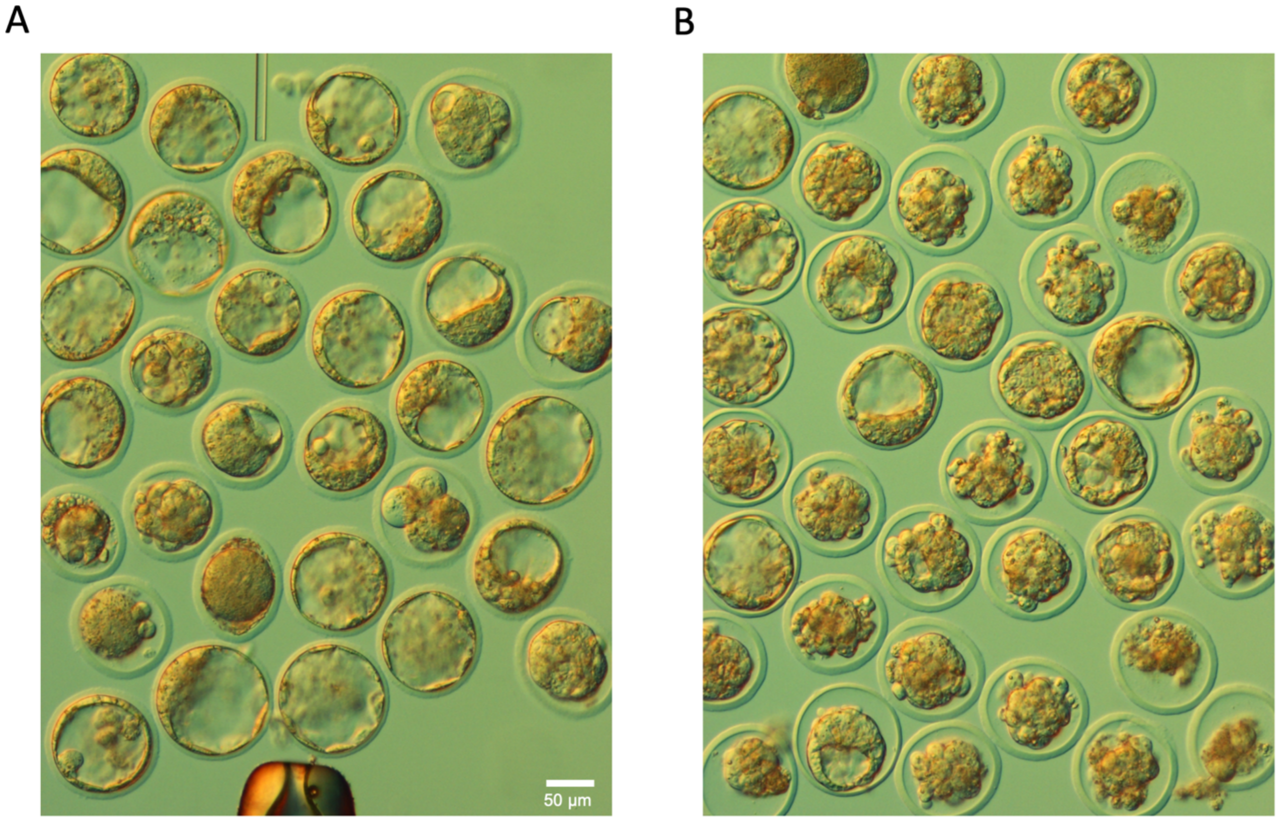

Supplementary Figure 2. Adverse effect of undefined cues emanating from the genital tract of stimulated females. Compared to blastocysts flushed from the uterus after natural ovulation and mating **(A)**, blastocysts flushed after gonadotropin stimulation present more often non-expanded cavities or collapsed cavities **(B)**.

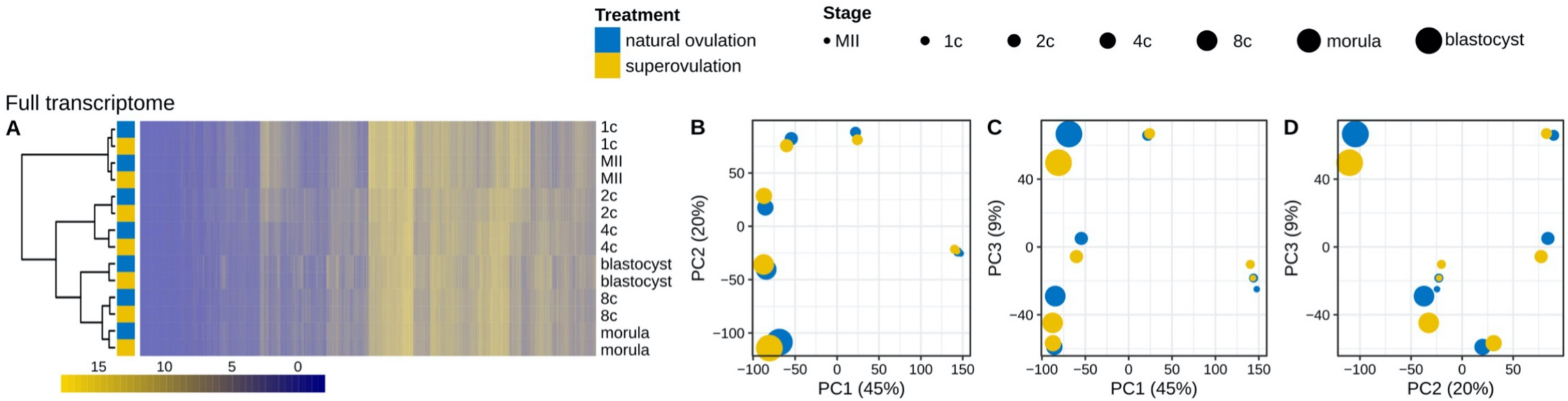

Supplementary Figure 3. Expression analysis of the full transcriptome (n=20,875). Hierarchical clustering **(A)** and principal component analysis **(B-D)** of the full transcriptome related to Figure 2. Samples were derived from pools of 210 oocytes or embryos, in duplicate for each treatment.

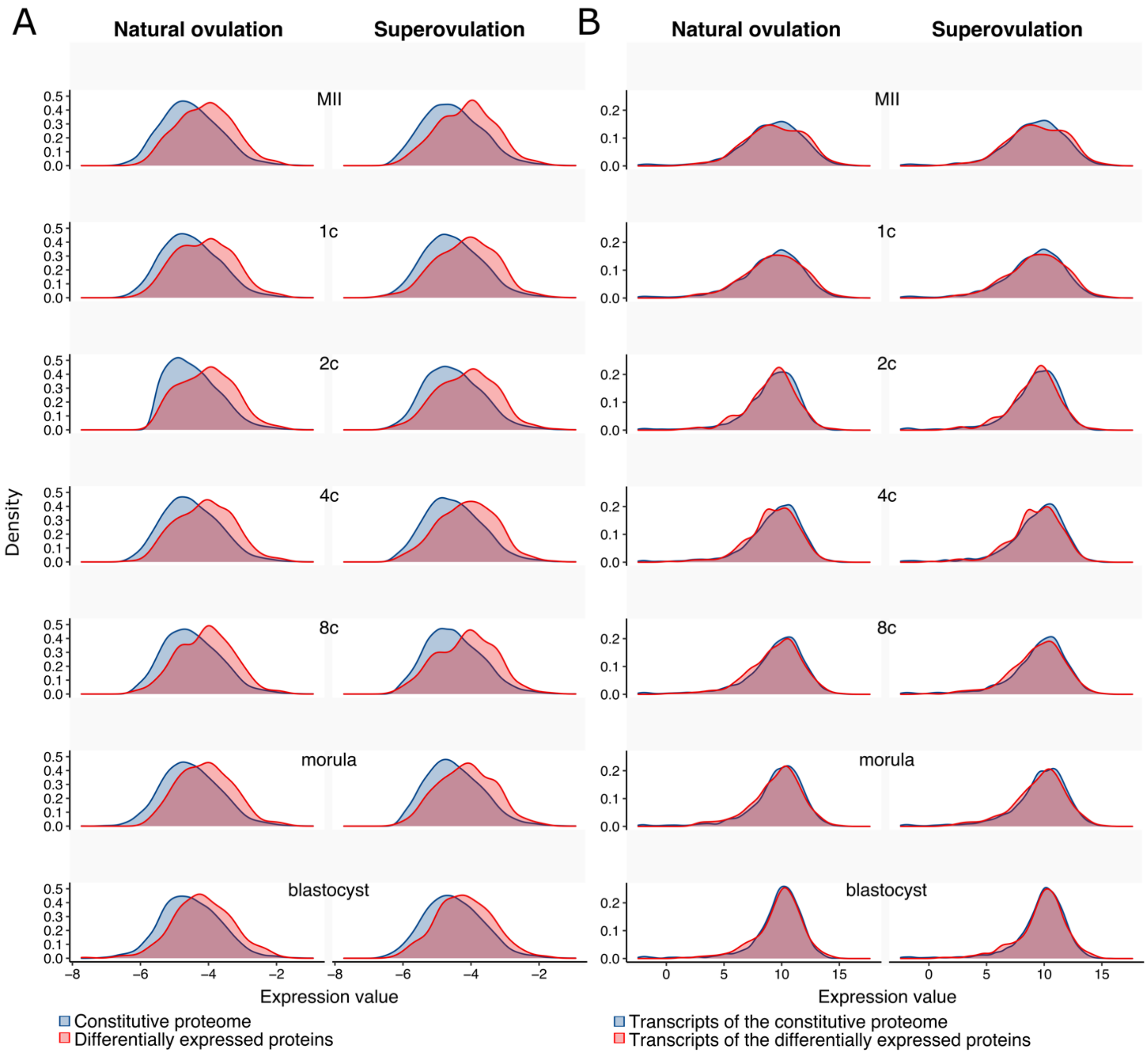

Supplementary Figure 4. Distribution of the expression values of the differentially expressed proteins and transcripts, compared to those of the 2,844 constitutively expressed proteins and their transcript counterparts, respectively (see Figure 2). Displayed is the probability density function (PDF) estimated using the density function of R; the curves can be employed to calculate approximately the probability of observing an expression value within a specific range. The differentially expressed proteins **(A)** are more highly expressed; this distinction cannot be seen in the transcriptome **(B).** "Expression values" are defined as described in the Materials and Methods section of the article.

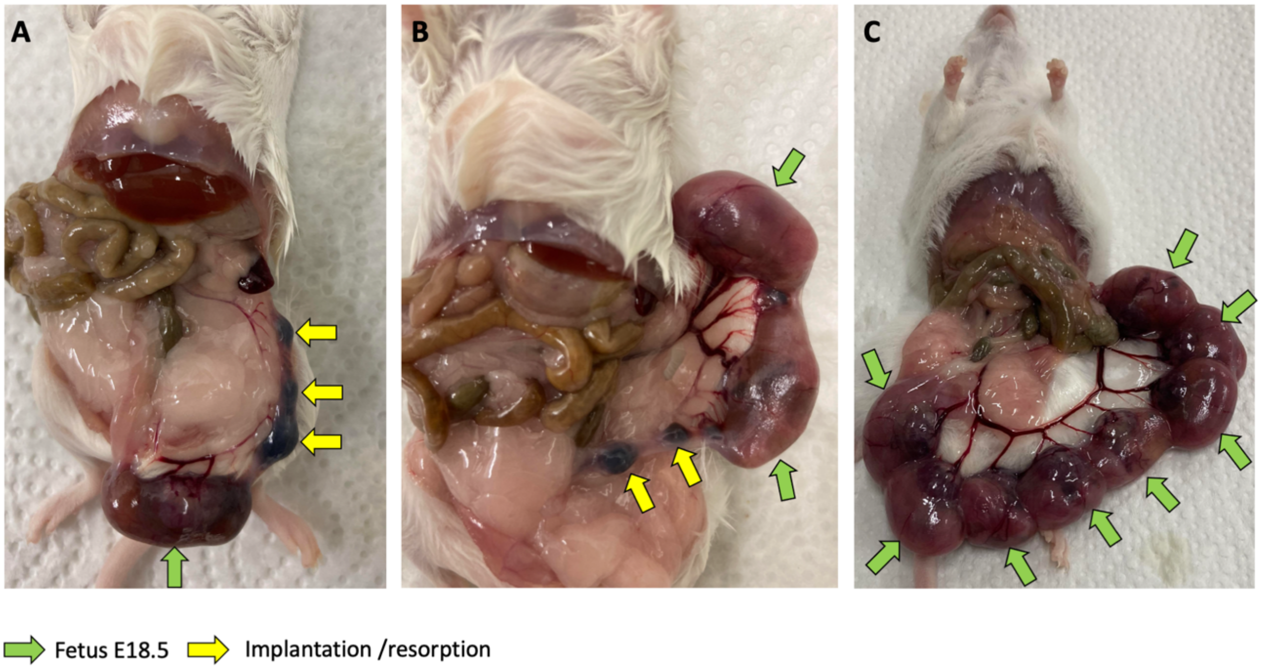

Supplementary Figure 5. Representative cases of hysterectomies conducted on day 18 after embryo transfer, related to Figure 5**. A**, case of a single fetus and 3 implantations (resorbed). **B**, case of 2 fetuses and 2 implantations (resorbed). **C**, case of 8 fetuses (no resorptions). A fixed number of 8 embryos was transferred pro recipient in all cases.

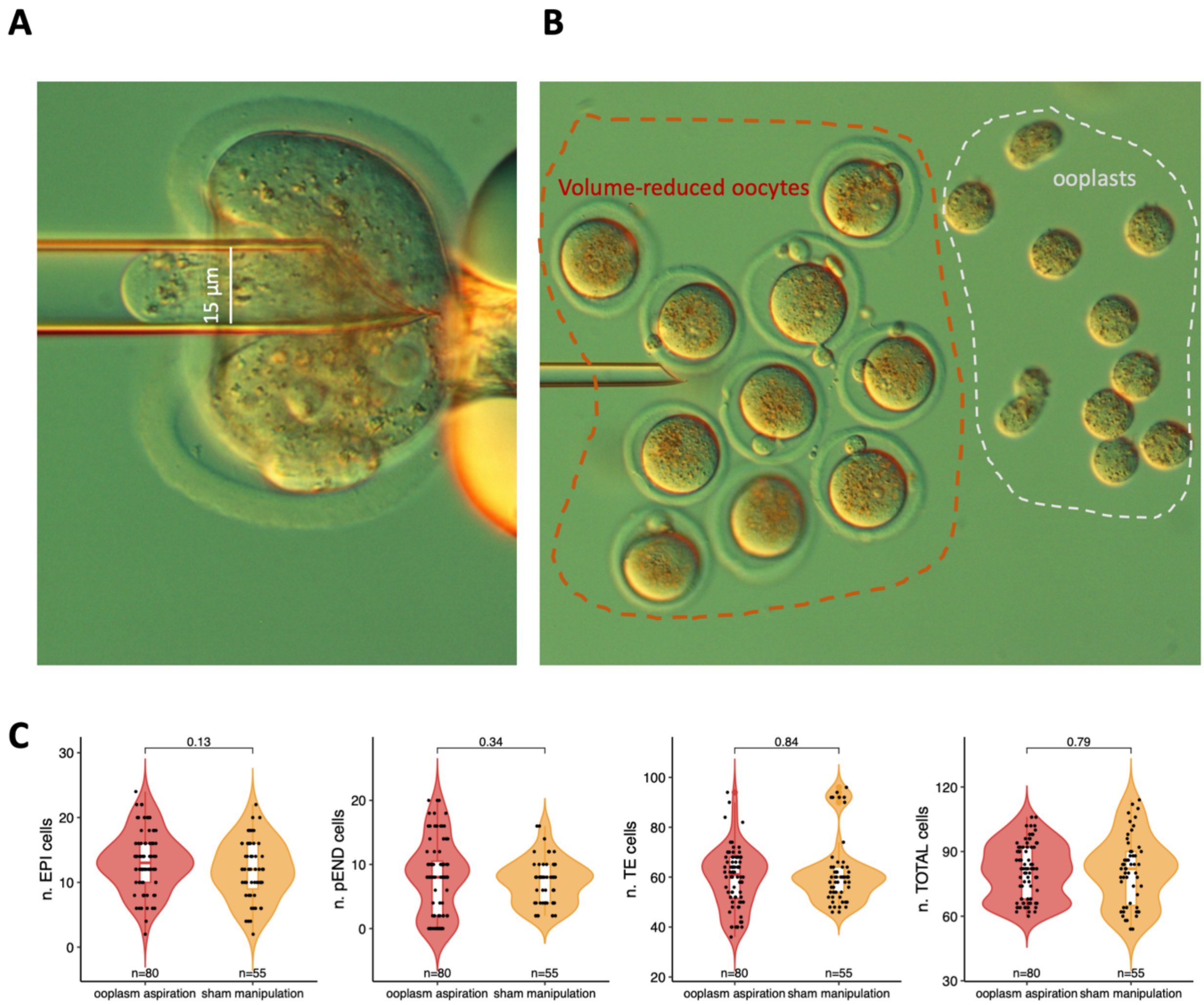

Supplementary Figure 6. Oocyte size *per se* is not causative of reduced embryonic fitness. (A, B) Ooplasm aspiration (-20%) does not exacerbate the deficit of primitive endoderm and epiblast **(C)** otherwise recorded in blastocysts derived from superovulated oocytes. Care was taken to not accidentally remove the pronuclei. Each data point corresponds to one blastocyst. P values refer to differences of cell number, in the particular blastocyst compartment, between groups (Wilcoxon test). Abbreviations: TE, trophectoderm; EPI, epiblast; pEND, primitive endoderm.

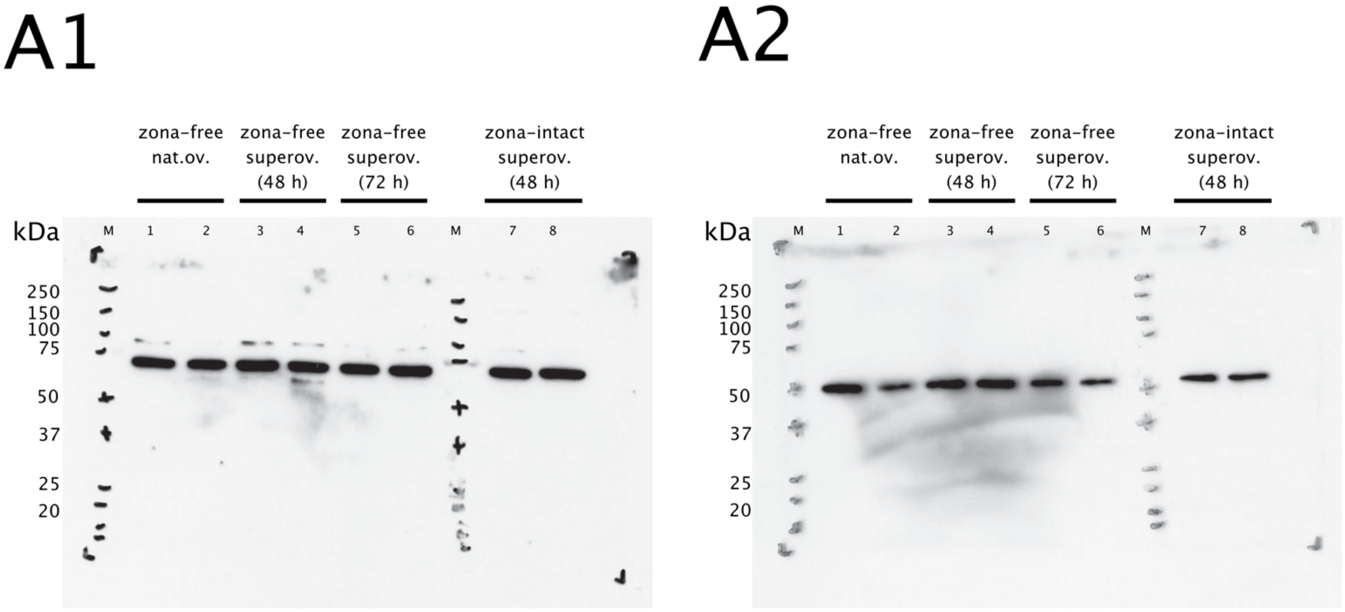

Lanes

M: Biorad Precision Plus Protein Prestained Protein Standard
1: #150 oocytes (zona free), natural
2: #149 oocytes (zona free), natural
3: #150 oocytes (zona free), 48 h
4: #150 oocytes (zona free), 48 h
5: #150 oocytes (zona free), 72 h
6: #150 oocytes (zona free), 72 h
7: #150 oocytes (with zona), 48 h
8. #150 oocytes (with zona), 48 h

Supplementary Figure 7. The uncropped Western blot images of Zp3 and α-tubulin shown in Supplementary Figure S1. Whole membrane of Zp3 protein detected with antibody PA5 89033 diluted 1:2000 **(A1).** Whole membrane of the loading control α-Tubulin detected with antibody T6199 diluted 1:5000 **(A2)**.

Supplementary Table S1: Dataset of 6444 proteins, SILAC/riBAQ$_P$ values (based on full data set PXD021331) of metaphase II mouse oocytes, 1-cell, 2-cell, 4-cell, 8-cell, morula and blastocyst-stage mouse embryos obtained after natural ovulation vs. super-ovulation (eCG and hCG 48 h apart). R1, R2 = replicates.
DOI: 10.6084/m9.figshare.14983689
https://figshare.com/s/30cbe76fb31a1aea360d

Supplementary Table S2: Transcript expression values of metaphase II mouse oocytes, 1-cell, 2-cell, 4-cell, 8-cell, morula and blastocyst-stage mouse embryos obtained after natural ovulation vs. super-ovulation (eCG and hCG 48 h apart). Dataset of 20875 transcripts, regularized log-transformed values obtained from normalized counts (based on full data sets DRA005956 and DRA006335). Expression values are obtained from the normalized counts by applying DESeq2's rlogTransformation() function with the "blind" parameter set to "TRUE" (i.e., without using any experimental design). Regularized log-transformed values were averaged across replicates.
DOI: 10.6084/m9.figshare.14983701
https://figshare.com/s/8335867a2030d7d820c0

Supplementary Table S3: Constitutive proteome: expression values of 2844 constitutive proteins always detected in metaphase II mouse oocytes, 1-cell, 2-cell, 4-cell, 8-cell, morula and blastocyst-stage mouse embryos obtained after natural ovulation vs. super-ovulation (eCG and hCG 48 h apart). Expression values are riBAQP values that were averaged across replicates, quantile-normalized, and log10 transformed.
DOI: 10.6084/m9.figshare.14983716
https://figshare.com/s/8957d917f2c9eb0a9987

Supplementary Table S4: Differential expression analysis of the constitutive proteome, comparing natural ovulation vs. super-ovulation (eCG and hCG 48 h apart).
DOI: 10.6084/m9.figshare.14983695
https://figshare.com/s/43fe3d958b05c9ef71d4

Supplementary Table S5: Differential expression analysis of the transcriptome, comparing natural ovulation vs. super-ovulation (eCG and hCG 48 h apart). Note that no P-value was computed for transcripts with a mean (log2-transformed) expression value across all samples below the 25th percentile.
DOI: 10.6084/m9.figshare.14983707
https://figshare.com/s/7706da5ad101d3676b00

Supplementary Table S6: Proteins describing the MP terms "thin zona pellucida", "embryonic growth arrest" and "absent inner cell mass proliferation", compared to housekeeping proteins. Expression values are riBAQP values, and their ranks.
DOI: 10.6084/m9.figshare.14983722
https://figshare.com/s/a01e4eed3a2c147f11b9

Supplementary Table S7: Proteome of metaphase II oocytes obtained after natural ovulation, super-ovulation (eCG and hCG 48 h apart), super-ovulation (eCG and

hCG 72 h apart), and in vitro maturation. Expression values are TMT values of 1857 proteins (based on full data set PXD026347). R1, R2, R3 = replicates.
DOI: 10.6084/m9.figshare.14983719
https://figshare.com/s/b95b0885fae22cdedc03
